# Chemical exposome patterns in mothers and children across urbanisation levels in five European birth cohorts

**DOI:** 10.1038/s41370-026-00859-6

**Published:** 2026-04-02

**Authors:** A. d’Errico, G. Moirano, C. Pizzi, M. Popovic, L. Chatzi, S. Andrusaityte, R. Grazuleviciene, R. Slama, R. McEachan, T.C., Yang, C. Thomsen, M. Vrijheid, L. Richiardi, M. Maule

**Affiliations:** 1 Cancer Epidemiology Unit, Department of Medical Sciences, University of Torino, Turin, Italy; 2 Institute for Risk Assessment Sciences (IRAS), Utrecht University, Utrecht, the Netherlands; 3 Department of Preventive Medicine, Keck School of Medicine, University of Southern California, Los Angeles, CA USA; 4 Department of Environmental Sciences, Vytautas Magnus University, Kaunas, Lithuania; 5 Team of Environmental Epidemiology, IAB, Institute for Advanced Biosciences, Inserm, CNRS, CHU-Grenoble-Alpes, University Grenoble-Alpes, Grenoble, France; 6 Bradford Institute for Health Research, Bradford Teaching Hospitals NHS Foundation Trust, Bradford, UK; 7 Department of Food Safety, Norwegian Institute of Public Health, Oslo, Norway; 8 ISGlobal, Barcelona, Spain; 9 Pompeu Fabra University, Barcelona, Spain; 10 CIBER de Epidemiología y Salud Pública (CIBERESP), Madrid, Spain

**Keywords:** Exposomics, Early Life Exposure, Biomonitoring, Child Exposure/Health, Exposure assessment, Maternal and foetal exposure/health, Perfluorinated, Chemicals, PFAS

## Abstract

**Background:**

Urbanisation can be an important determinant of human exposure to synthetic chemical pollutants. The impact of contaminant exposures on health is of particular concern during susceptible periods of life, such as in utero and during childhood, when exposure may lead to adverse health effects in childhood and later adulthood.

**Objective:**

We aimed to examine how contaminant exposures vary between urban and non-urban areas across five different European birth cohorts in Spain, France, Greece, the UK, and Lithuania.

**Methods:**

Urine and blood samples were collected from a total of 1021 mother-child pairs during both pregnancy and childhood (6–11 years old). Concentration levels of forty metabolites—including PFASs, phenols, phthalates, metals, and organophosphate and persistent pesticide metabolites—were measured. We used a spatial indicator to define the participants’ degree of urbanisation. Linear Mixed-Effect Models were used to compare the distribution of exposures between urban and non-urban areas for the two life stages separately.

**Results:**

The concentrations of contaminants varied by degree of urbanisation and life stage. Overall, concentrations of phenols (GMRs; Geometric Mean Ratios, ranging from 1.06 to 1.56) and PCBs (GMRs ranging from 1.07 to 1.15) were higher among pregnant mothers living in urban areas compared to those in non-urban areas. Children showed more heterogeneous patterns of exposure across contaminant families. Children in urban areas had lower concentration levels of PFASs (GMRs ranging from 0.84 to 0.97) but higher concentration levels of phenols (GMRs ranging from 1.05 to 1.15) and phthalates (GMRs ranging from 1.05 to 1.17) compared to those in non-urban areas.

**Significance:**

Our study contributes to the understanding of how the degree of urbanisation characterises children’s exposure to hazardous substances. Our findings align with the existing literature, which shows varying profiles of environmental exposures based on different degrees of urbanisation.

**Impact:**

Our study provides important insights into how the degree of urbanisation can influence children’s exposure to hazardous substances during critical developmental windows, with potential implications for both immediate and long-term health outcomes. Specifically, phenols, phthalates, and PCBs were found to be more prevalent in individuals living in urban areas, with notable heterogeneity of PCB concentrations across European cohorts. In contrast, PFAS concentrations were higher in children residing in non-urban areas. Understanding the geographic variations in exposure to hazardous contaminants is useful for identifying areas with higher contaminant levels, which may have important implications for vulnerable populations.


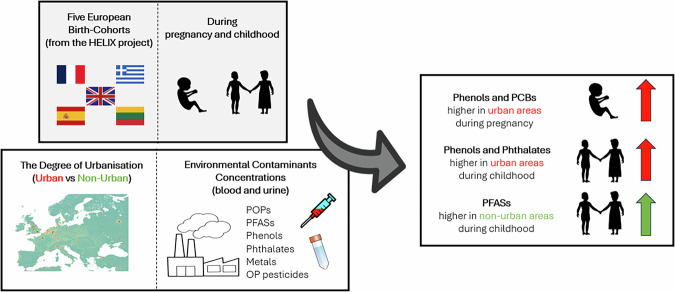


## Introduction

Humans are exposed daily to an unquantifiable number of synthetic chemicals. The prevalence of hazardous chemicals in the environment is disconcerting, with estimates ranging from 23,000 to over 68,000 substances [1]. Although many of these have contributed to the development of modern societies, particularly in the Western world, their pervasive presence raises concerns regarding potential adverse effects on the environmental ecosystem and human health [2], particularly during critical developmental periods such as during foetal and early postnatal life and in childhood [3]. Children may be more susceptible than adults to the potential negative impacts of contaminants due to distinct physiological characteristics and a higher risk of exposure. Factors contributing to this increased susceptibility include a higher body surface area-to-weight ratio, which facilitates dermal absorption, elevated respiratory rates and minute ventilation leading to greater inhalation exposure, and increased intake of water and food per unit of body weight [4]. According to the Developmental Origins of Health and Disease (DOHAD) hypothesis, exposure to substances during foetal development can lead to lasting alterations in the body’s structure, physiology, and metabolism [5]. The health and well-being of societies, as well as the prospects for future generations, depend on the healthy and unhindered progression through these developmental stages [6]. In the European Union alone, around 167 million tonnes of hazardous chemicals were produced in 2023 [7]. Around 40 million tons of carcinogenic, mutagenic, and reprotoxic chemicals were produced in the European Union every year between 2004 and 2023 [7]. Given these numbers, the existing European regulations, designed to protect individuals of all ages, may prove inadequate in the face of such pervasive and potentially harmful substances present in the environment. For example, compounds like phthalates and parabens, commonly employed in plastics and personal care products, and per- and polyfluoroalkyl substances (PFASs), recognised for their chemical and thermal stability, have been linked to an elevated risk of developmental disabilities such as autism, cerebral palsy, and various forms of mental retardation [8]. These compounds have also been associated with chronic conditions like asthma, allergies, diabetes, and obesity [9, 10]. The distribution of contaminants is likely to differ across urban, suburban, and rural areas. Over 25% of the European population resides in rural areas, and when rural areas are combined with suburban ones, this proportion exceeds 50% [7, 11]. This highlights the importance of assessing contaminant distribution to identify areas where concentrations may be of concern. Through this research, we aim to explore how the degree of urbanisation could shape children’s exposure to environmental contaminants during developmental years. In particular, we assessed the distribution of exposure to six distinct families of substances, known for their impact on children’s health, including persistent pesticides and organophosphate (OP) pesticides, PFASs, phthalates, phenols, and heavy metals. We focused on critical developmental periods, specifically during pregnancy, referring to maternal exposures occurring during foetal development, and childhood (ages 6–11 years), and included participants from five European birth cohorts, providing a comprehensive analysis of the exposure patterns in urban and non-urban environments.

## Methods

### Study population and samples collection

The “Human Early Life Exposome” (HELIX) study, a European population-based exposome investigation, has been described in a previous publication [12]. In our research, we focused on participants of five HELIX mother-child cohorts: BiB (Born in Bradford, UK, *n* = 205) [13], EDEN (Study of Determinants of Pre- and Postnatal Development, France, *n* = 198) [14], INMA (Environment and Childhood, Spain, *n* = 223) [15], KANC (Kaunas Cohort, Lithuania, *n* = 196) [16], and RHEA (Mother–Child Cohort in Crete, Greece, *n* = 199) [17]. Of the 1301 mother-child pairs with available biomarker data, 280 were excluded due to missing residential address information, leaving 1021 pairs for analysis. A standardised protocol was used for the collection of urine and blood samples from pregnant women and from their children aged 6 to 11 years during the follow-up examination [12]. No urine samples were collected from KANC participants.

### Degree of urbanisation indicator

We assigned the degree of urbanisation to each participant, based on their residential address information, using the Global Human Settlement-Settlement Model (GHS-SMOD) raster, which classifies regions along the urban-rural spectrum with 1 km resolution [18]. The GHS-SMOD raster is obtained by integrating three key factors: population size, population density, and built-up area density per square kilometre [19]. An urban centre consists of contiguous grid cells with a density of at least 1500 inhabitants per km² and a collective population of at least 50,000. A rural cluster consists of contiguous grid cells with a density of at least 300 inhabitants per km² and a collective population between 500 and 4999. A suburban cluster consists of contiguous grid cells with a density of at least 900 inhabitants per km² and a collective population of at least 2500, provided that the cluster is not within 2 km of a dense urban cluster or an urban centre. The resulting settlement typologies derived from the degree of urbanisation indicator encompass urban centres, peri-urban areas (e.g., dense urban clusters, semi-dense urban clusters, and suburban grid cells), and rural areas (e.g., rural clusters, low-density rural grid cells, and very low-density grid cells). Given the low prevalence of participants living in peri-urban and rural settlements, we grouped residents of these two settlements into a single category, denoted non-urban.

### Environmental contaminants

The procedures for the collection and analysis of biological samples of women during pregnancy and children (6-11 years) are described in detail in Maitre et al. and Haug et al. [12, 20]. The maternal samples from the various cohorts were collected at different time points: INMA, KANC, and RHEA during the first trimester; EDEN during the first and second trimesters; and BIB during the second and third trimesters. To reduce measurement uncertainty and ensure comparability, most measurements for each chemical exposure biomarker were performed in a single laboratory. The samples were randomised into batches prior to chemical analyses, with each batch including samples from at least three cohorts. The assessments of all children were conducted in a fully harmonised manner, using the same protocols for sample collection and clinical examination across all six cohorts. The assessments of children were conducted between December 2013 and February 2016 [21]. Measured environmental contaminants can be grouped according to their chemical characteristics into six distinct families: (i) persistent pesticides, including organochlorine compounds (DDE, DDT, HCB), polychlorinated biphenyls (PCB-118, -138, −153, −170, −180), polybrominated diphenylethers (PBDE-47, -153); (ii) per- and polyfluoroalkyl substances (PFASs: PFOA, PFNA, PFUnDA, PFHxS, PFOS); (iii) metals and elements (As, Cd, Hg, Pb) in blood; (iv) phthalate metabolites (MEP, MiBP, MnBP, MBzP, MEHP, MEHHP, MEOHP, MECPP, oh-MiNP, oxo-MiNP, sumDEHP); (v) phenols (MEPA, ETPA, PRPA, BPA, BUPA, OXBE, TCS); and (vi) non-specific OP pesticide metabolites (DMP, DMTP, DEP, DETP) in urine. The lipophilic compounds, such as organochlorine compounds and PBDEs, were adjusted for the total lipid content in the blood and expressed in ng/g of lipids. Compounds analysed in urine samples were adjusted for creatinine and expressed in μg/g of creatinine. Methods used to address the limit of detection (LOD) were reported in the article by Haug et al. (2018) [20], which characterised the chemical exposome data of the HELIX project, specifically in the supplementary materials (“Excel Table 5” therein). In total, 39 metabolites were measured during pregnancy and 41 among children. PBDE-47 and PBDE-157 were only included in the children’s analysis because their concentrations in pregnant women were detectable in less than 80% of samples, which was the inclusion threshold for all contaminants and related metabolites. Absolute concentration levels of contaminants in urban and non-urban areas are shown as geometric means (GM) and standard deviations in Tables 1 and 2 for pregnant women and children, respectively.

**Table 1:** Biomonitoring concentrations of environmental contaminants during pregnancy in urban and non-urban areas.

Pregnancy								
Chemical contaminant	Abbrev	Unit	Urban			Non-urban		
			N	GM	GSD	N	GM	GSD
**Persistent pesticides (blood samples)**								
4,4′dichlorodiphenyldichloroethylene	DDE	ng/g lipid	704	64.4	4.0	299	78.3	4.2
4,4′dichlorodiphenyltrichloroethane	DDT	ng/g lipid	704	1.5	3.7	299	2.69	2.8
Hexachlorobenzene	HCB	ng/g lipid	704	10.3	2.4	299	8.5	2.0
2,3′,4,4′,5-Pentachlorobiphenyl	PCB-118	ng/g lipid	704	1.7	1.9	299	4.1	2.4
2,2′,3,4,4′,5′-Hexachlorobiphenyl	PCB-138	ng/g lipid	704	6.9	2.1	299	14.0	2.3
2,2′,4,4′,5,5′-Hexachlorobiphenyl	PCB-153	ng/g lipid	704	13.2	2.0	299	27.3	2.4
2,2′,3,3′,4,4′,5-Heptachlorobiphenyl	PCB-170	ng/g lipid	704	1.9	2.7	299	7.4	2.9
2,2′,3,3′,4,4′,5-Heptachlorobiphenyl	PCB-180	ng/g lipid	704	7.4	2.5	299	18.2	2.8
**Per- and Polyfluoroalkyl Substances (blood samples)**								
Perfluorooctanoate	PFOA	μg/L	714	1.9	2.1	307	2.8	1.8
Perfluorononanoate	PFNA	μg/L	714	0.6	2.6	307	1.0	1.8
Perfluoroundecanoate	PFUnDA	μg/L	714	0.1	3.0	307	0.2	1.9
Perfluorohexane sulphonate	PFHxS	μg/L	714	0.5	2.3	307	0.7	2.3
Perfluorooctane sulphonate	PFOS	μg/L	714	4.5	2.1	307	8.9	2.0
**Metals (blood samples)**								
Arsenic	As	μg/L	713	0.6	3.2	307	0.8	3.1
Cadmium	Cd	μg/L	713	0.3	2.2	307	0.3	2.2
Mercury	Hg	μg/L	713	1.7	2.8	307	2.2	2.2
Lead	Pb	μg/L	713	10.4	1.7	307	11	1.6
**Phthalate Metabolites (urine samples)**								
Monoethyl phthalate	MEP	μg/g creat	714	236	3.4	307	151	3.4
Mono-iso-butyl phthalate	MiBP	μg/g creat	714	36.5	2.1	307	50.2	2.2
Mono-n-butyl phthalate	MnBP	μg/g creat	714	26.5	2.3	307	38.9	2.7
Mono-n-butyl phthalate	MBzP	μg/g creat	713	6.3	3.0	307	14.3	3.6
Mono-2-ethylhexyl phthalate	MEHP	μg/g creat	702	6.5	2.6	301	8.7	2.8
Mono-2-ethyl-5-hydroxyhexyl phthalate	MEHHP	μg/g creat	714	16.0	2.4	307	25.6	2.6
Mono-2-ethyl-5-oxohexyl phthalate	MEOHP	μg/g creat	714	12.7	2.3	307	18.7	2.7
Mono-2-ethyl-5-carboxypentyl phthalate	MECPP	μg/g creat	714	32.4	2.0	307	41.3	2.2
Sum of Di(2-ethylhexyl)phthalate	sumDEHP	μg/g creat	714	12.7	1.3	307	14.4	1.3
Mono-4-methyl-7-hydroxyoctyl phthalate	oh-MiNP	μg/g creat	714	0.8	2.6	307	0.6	3.6
Mono-4-methyl-7-oxooctyl phthalate	oxo-MiNP	μg/g creat	714	1.0	2.7	307	0.7	3.3
**Phenols (urine samples)**								
Methyl paraben	MEPA	μg/g creat	714	162	6.6	307	92.1	6.0
Ethyl paraben	ETPA	μg/g creat	713	6.6	8.6	306	4.4	5.6
Propyl paraben	PRPA	μg/g creat	703	27.8	12.1	303	12.2	11.0
Bisphenol-A	BPA	μg/g creat	705	2.3	2.9	306	2.3	2.3
N-Butyl paraben	BUPA	μg/g creat	712	1.4	11.7	306	1.3	8.7
Oxybenzone	OXBE	μg/g creat	714	7.0	8.3	307	3.0	6.4
Triclosan	TCS	μg/g creat	714	11.8	9.6	307	14.0	12.1
**OP Pesticide metabolites (urine samples)**								
Dimethyl phosphate	DMP	μg/g creat	711	6.0	3.9	306	8.8	3.2
Dimethyl thiophosphate	DMTP	μg/g creat	713	2.7	8.0	307	5.4	4.7
Diethyl phosphate	DEP	μg/g creat	712	3.1	2.9	307	3.5	2.6
Diethyl thiophosphate	DETP	μg/g creat	700	0.5	6.0	301	0.6	5.9

*N* number of subjects, *GM* geometric mean, *GSD* geometric standard deviation, *creat* creatinine.

**Table 2:** Biomonitoring concentrations of environmental contaminants during childhood in urban and non-urban areas.

Childhood								
Chemical contaminant	Abbrev	Unit	Urban			Non-urban		
			N	GM	GSD	N	GM	GSD
**Persistent pesticides (blood samples)**								
4,4′dichlorodiphenyldichloroethylene	DDE	ng/g lipid	704	26.8	2.9	299	25.0	2.8
4,4′dichlorodiphenyltrichloroethane	DDT	ng/g lipid	704	0.5	5.8	299	0.7	4.4
Hexachlorobenzene	HCB	ng/g lipid	704	9.3	1.8	299	7.2	1.6
2,2′,4,4′-Tetrabromodiphenyl Ether	PBDE-47	ng/g lipid	704	0.2	3.6	299	0.2	2.8
2,2′,4,4′,5,5′-Hexabromodiphenyl ether	PBDE-153	ng/g lipid	704	0.1	4.5	299	0.1	4.4
2,3′,4,4′,5-Pentachlorobiphenyl	PCB-118	ng/g lipid	704	2.0	1.9	299	2.2	1.6
2,2′,3,4,4′,5′-Hexachlorobiphenyl	PCB-138	ng/g lipid	704	4.7	2.0	299	5.0	1.9
2,2′,4,4′,5,5′-Hexachlorobiphenyl	PCB-153	ng/g lipid	704	2.3	1.8	299	10.8	1.7
2,2′,3,3′,4,4′,5-Heptachlorobiphenyl	PCB-170	ng/g lipid	704	0.7	3.9	299	1.0	3.0
2,2′,3,3′,4,4′,5-Heptachlorobiphenyl	PCB-180	ng/g lipid	704	2.6	3.0	299	3.3	2.4
**Per- and Polyfluoroalkyl Substances (blood samples)**								
Perfluorooctanoate	PFOA	μg/L	714	1.4	1.4	307	1.4	1.4
Perfluorononanoate	PFNA	μg/L	714	0.3	2.1	307	0.4	1.7
Perfluoroundecanoate	PFUnDA	μg/L	714	0	2.6	307	0	2.7
Perfluorohexane sulphonate	PFHxS	μg/L	714	0.2	2.3	307	0.3	2.6
Perfluorooctane sulphonate	PFOS	μg/L	714	1.4	2.2	307	2.5	1.8
**Metals (blood samples)**								
Arsenic	As	μg/L	713	1.2	3.3	307	1.1	3.1
Cadmium	Cd	μg/L	713	0	3.3	307	0	4.1
Mercury	Hg	μg/L	713	0.7	3.7	307	1.0	2.9
Lead	Pb	μg/L	713	9.0	1.5	307	9.6	1.5
**Phthalate Metabolites (urine samples)**								
Monoethyl phthalate	MEP	μg/g creat	714	52	2.9	307	40	2.8
Mono-iso-butyl phthalate	MiBP	μg/g creat	714	52	2.0	307	45	2.1
Mono-n-butyl phthalate	MnBP	μg/g creat	714	26	2.1	307	22	2.1
Mono-n-butyl phthalate	MBzP	μg/g creat	713	5.2	2.4	307	6.4	2.2
Mono-2-ethylhexyl phthalate	MEHP	μg/g creat	702	3.6	2.3	301	2.7	2.4
Mono-2-ethyl-5-hydroxyhexyl phthalate	MEHHP	μg/g creat	714	27	1.9	307	18	2.2
Mono-2-ethyl-5-oxohexyl phthalate	MEOHP	μg/g creat	714	16	1.9	307	11	2.2
Mono-2-ethyl 5-carboxypentyl phthalate	MECPP	μg/g creat	714	47	1.9	307	30	2.2
Sum of Di(2-ethylhexyl)phthalate	sumDEHP	μg/g creat	714	88	1.9	307	59	2.2
Mono-4-methyl-7-hydroxyoctyl phthalate	oh-MiNP	μg/g creat	714	6.6	1.9	307	4.7	2.1
Mono-4-methyl-7-oxooctyl phthalate	oxo-MiNP	μg/g creat	714	3.4	2.2	307	2.6	2.2
**Phenols (urine samples)**								
Methyl paraben	MEPA	μg/g creat	714	15	6.3	307	8.5	5.1
Ethyl paraben	ETPA	μg/g creat	713	0.9	3.7	306	0.7	3.1
Propyl paraben	PRPA	μg/g creat	703	0.5	18.7	303	0.1	16.2
Bisphenol-A	BPA	μg/g creat	705	4.8	2.7	306	3.0	2.6
N-Butyl paraben	BUPA	μg/g creat	712	0.1	3.3	306	0.1	3.1
Oxybenzone	OXBE	μg/g creat	714	3.2	5.3	307	1.3	5.0
Triclosan	TCS	μg/g creat	714	1.1	3.7	307	0.8	4.5
**OP Pesticide metabolites (urine samples)**								
Dimethyl phosphate	DMP	μg/g creat	711	1.0	4.7	306	1.2	5.0
Dimethyl thiophosphate	DMTP	μg/g creat	713	2.4	4.9	307	2.6	4.6
Diethyl phosphate	DEP	μg/g creat	712	1.3	6.5	307	1.1	6.1
Diethyl thiophosphate	DETP	μg/g creat	700	0.3	5.3	301	0.4	5.9

*N* number of subjects, *GM* geometric mean, *GSD* geometric standard deviation, *creat* creatinine.

### Statistical analysis

All contaminants’ measurements were log2-transformed to approximate normality, and their distributions were summarised using the GM and geometric standard deviation (GSD). To compare and visualise exposure distributions for each chemical and across the degree of urbanisation during pregnancy and childhood, probability density functions are shown in Fig. 2S–13S.

Linear mixed-effects models (LMEMs) with random intercepts varying by cohort were used to assess the distribution of exposure levels to environmental contaminants during pregnancy and childhood across the two urbanisation classes considered (non-urban and urban). A non-urban area was used as the reference in the analysis. LMEMs were fitted separately per contaminant, and results were reported as geometric mean ratio (GMR), which can be interpreted as a measure of the relative magnitude of contaminant concentrations in urban vs non-urban participants. The Wald 95% confidence intervals (CI) were estimated. The Intraclass Correlation Coefficient (ICC) was used to quantify the proportion of variance due to the between-cohort heterogeneity. The ICC is calculated as the ratio of the between-cohorts variance over the total variance, given by the sum of the between- and the within-cohort variances [21]. For five of the six contaminant families—persistent pesticides, per- and polyfluoroalkyl substances (PFAS), phthalates, phenols, and OP pesticide metabolites—we conducted a within-family Principal Component Analysis (PCA) to summarise exposure profiles separately for pregnancy and childhood (*prcomp* R function). Metals were excluded from this step because they were less correlated and represented a more heterogeneous group of compounds. PCA identifies linear combinations of the measured contaminants that capture the main patterns of variation within each family. For each family, we retained the first principal component (PC1), which explains the largest proportion of variance. Each mother and child was assigned a PC1 score, calculated as a weighted sum of all contaminants in the family, with weights derived from the PCA. The PC1 score represents an overall summary of exposure within that family: higher scores indicate greater exposure to the combination of chemicals characterising that family. These PC1 scores were then used as outcome variables in LMEMs to examine associations with the degree of urbanisation. Within each of the five groups, the first principal component was derived for the same environmental contaminants for both mothers and children. The sole exception was the component related to the persistent pesticides’ family, where the principal component for mothers was derived, excluding PBDE-47 and PBDE-153 because of their high percentage below the LOQ (Limit of Quantification).

For the analyses of children, we adjusted the LMEMs for sex to account for male–female metabolic differences [22]. Other potential confounding factors were intentionally omitted from the analyses because the degree of urbanisation was considered a distal determinant of overall environmental exposures. In addition, the analysis was primarily descriptive, with no intention of drawing causal conclusions or generating hypotheses about the reasons for potential variations in environmental concentration distributions across different urbanisation levels.

Lastly, Pearson correlation coefficients were calculated between the two periods of chemical exposure levels assessment (during pregnancy and childhood) to assess the extent to which exposures in mothers and children, who generally share the same environment, are correlated.

### Research in context

To facilitate comparison of our results with the existing literature, we searched PubMed for relevant studies and classified them, including our own, according to whether they reported higher, lower or similar concentrations of environmental contaminants in urban versus non-urban areas. In particular, we searched PubMed in April 2024 using the terms “degree of urbanisation,” “urban,” “rural,” “chemicals,” “exposure,” “pregnancy,” “children,” and “childhood,” without restrictions on date or language. Based on title and abstract screening and full-text review, thirty-five papers were selected: ten on persistent pesticides, four on PFAS, six on metals, five on OP pesticides, six on phthalates, and four on phenols. Studies were included if they performed biomonitoring of these substances in urban and non-urban areas.

## Results

### Degree of urbanisation and single environmental contaminants

Tables 1 and 2 show the distributions of environmental contaminants measured during pregnancy and childhood, respectively, summarised by GMs and GSDs for participants living in urban and non-urban areas. These tables provide absolute concentration data that serve as reference values and illustrate exposure variability across settings. To complement these summaries, probability density functions showing the full distributions by degree of urbanisation are provided in Supplementary Figs. S2–S13.

**Fig. 1:**
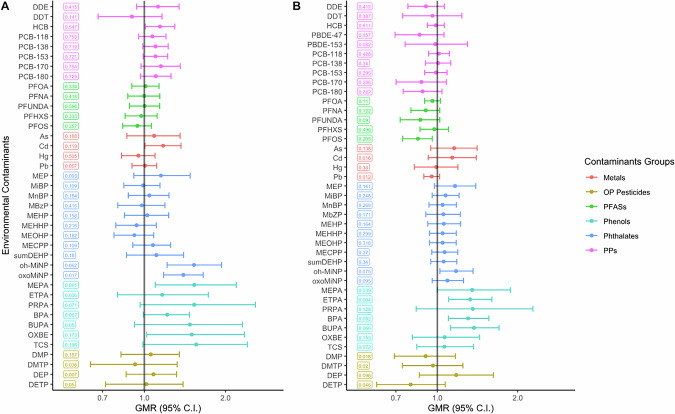
Associations between the degree of urbanisation and the chemical exposome of mothers and children. **A** GMRs for the association between the degree of urbanization and exposure to environmental contaminants during pregnancy. **B** GMRs (95% CI) for the association between the degree of urbanisation and exposure to environmental contaminants among children (6–11 yrs). For interpretation: GMR values < 1 indicate lower exposure and GMR values > 1 indicate higher exposure in the subjects living in urban areas in comparison to the reference category (non-urban areas). GMR Geometric Mean Ratio, C.I. Confidence Interval, OP Organophosphate, PFASs Per- and polyfluoroalkyl substances, PPs Persistent Pesticides.

Given the variability observed across contaminants and populations, Tables 1 and 2 are intended to characterise exposure levels rather than to directly infer differences between urban and non-urban groups. Formal statistical comparisons of urban versus non-urban concentrations are therefore presented using GMRs, which provide a rigorous assessment of exposure contrasts between groups. These results are presented in Fig. 1. Overall, pregnant women (Fig. 1A) residing in urban areas showed higher contaminant concentrations than those in non-urban areas, particularly for persistent pesticides and phenols. The highest increased levels were observed for phenols (GMRs ranging from 1.06 to 1.56), for which there was low variability between cohorts. All PCBs (GMRs ranging from 1.07 to 1.15) and DDE (GMR 1.12; 95% CI 0.93, 1.35) showed relatively higher concentration among mothers living in urban areas and exhibited considerable variability between cohorts. For metals, we observed a relevant difference between urban and non-urban areas only for cadmium, with 17% higher concentrations in the urban group. Phthalate concentrations, with GMRs ranging from 0.92 to 1.49, were similar between the two areas. However, levels of oh-MiNP (GMR 1.53, 95% CI 1.21,1.93) and oxoMiNP (GMR 1.40, 95% CI 1.18,1.65) were considerably higher in urban areas. OP pesticide metabolites (GMRs ranging from 0.92 to 1.08) and PFASs (GMRs ranging from 0.94 to 1.01) were relatively similar among women living in non-urban and urban areas.

Similarly, children (Fig. 1B) residing in urban areas appeared to be more exposed to phenols than those in non-urban areas and low heterogeneity between cohorts was observed. For example, BUPA (GMR 1.37; 95% CI 1.12, 1.70), ETPA (GMR 1.33; 95% CI 1.10, 1.56) and BPA (GMR 1.30; 95% CI 1.10, 1.56) concentrations were more than 30% higher among children of urban than non-urban areas. Phthalate compounds also showed higher concentrations in children residing in urban areas, although to a smaller extent (GMRs ranging from 1.05 to 1.17), with oh-MiNP exhibiting the largest difference. In contrast, all PFASs compounds showed lower concentrations in children residing in urban areas compared to those in non-urban areas, with GMRs between 0.84 and 0.97. The most notable difference in exposure between urban and non-urban children was observed for PFOS (GMR 0.84, 95% CI 0.74, 0.96). Similarly, those living in urban areas had lower concentrations of some persistent pesticides (PBDE-47: GMR 0.85, 95% CI 0.69, 1.05, and PCB-170: GMR 0.87, 95% CI 0.70, 1.08) and OP pesticides (in particular, DETP: GMR 0.79, 95% CI 0.59, 1.07), with the exception of DEP.

PCBs showed high between-cohort heterogeneity during pregnancy (ICC ≈ 0.7), whereas heterogeneity was much lower for all other compounds, particularly for phenols and OP pesticides in both periods. Among children, heterogeneity was overall low across all compounds.

**Fig. 2:**
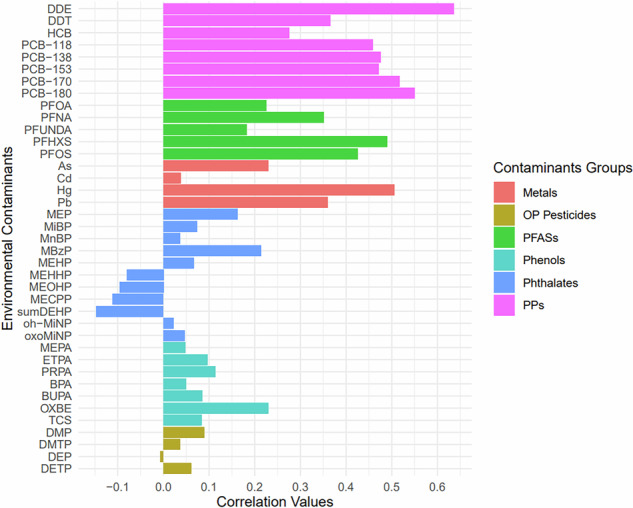
Pearson correlation coefficients for chemical levels between mothers and children. The coefficient’s value ranges from +1 (perfect positive correlation) to −1 (perfect negative correlation), with 0, on the other hand, indicating no correlation. OP organophosphate, PFASs per- and polyfluoroalkyl substances, PPs persistent pesticides.

Figure 2 shows the correlation between contaminant levels measured during pregnancy and childhood. The highest correlations were observed for DDE (*r* = 0.63) and PCBs (from 0.45 to 0.55). Mercury (Hg), PFHxS, and PFOS also showed relatively strong correlations (*r* = 0.51, 0.49, and 0.43, respectively), while many phthalates, OP pesticides, and phenols exhibited little to no correlation between the two periods.

### Degree of urbanisation and components combining environmental contaminants

The first components extracted from the PCA provide an overview of the exposure to the five families of contaminants analysed (Table 3). In pregnant women, the first component of the persistent pesticides family accounted for a significant proportion (64%) of the total variance, with high loadings of PCB-180 (0.60), PCB-138 (0.50) and PCB-153 (0.48); the first component of PFASs explained 72% of the total variance, and was highly loaded of PFNA (0.53), PFHxS (0.52), and PFOS (0.51); 50% of the total variance was explained by the first component of phthalates with high loadings on sumDEPH (0.41), MEOHP (0.41), and MEHHP (0.40); the first component of phenols explained 46% of the total variance and was highly loaded of PRPA (0.57), BUPA (0.49) and ETPA (0.43); lastly, the first component of OP pesticide metabolites explained 56% of the total variance and was highly loaded of DMDTP (0.60) and DMTP (0.59).

**Table 3:** Association between degree of urbanisation (categorised as non-urban and urban) and the first principal component derived from principal-component analysis for each chemical family during pregnancy and childhood (6–11 years).

Pregnancy		Childhood	
Component Family	Beta (95% CI) (ref: non-urban)	Component Family	Beta (95% CI) (ref: non-urban)
Persistent pesticides	0.22 (0.01–0.44)	Persistent pesticides	−0.18 (–0.52 to 0.16)
PFAS	−0.04 (–0.28 to 0.18)	PFASs	−0.23 (−0.45 to −0.01)
Phthalates	–0.30 (−0.73 to 0.17)	Phthalates	0.26 (−0.10 to 0.63)
Phenols	0.81 (0.11–1.53)	Phenols	0.53 (−0.03 to 1.12)
OP pesticide metabolites	0.14 (−0.43 to 0.68)	OP pesticide metabolites	−0.07 (−0.34 to 0.22)

The relationship between the degree of urbanisation and exposure to different contaminant families was also investigated using LMEMs fitted to PC1 scores for each contaminant family. The results are expressed as regression coefficients (β), which are unitless because the PC1 scores are themselves standardised combinations of contaminant levels. A positive β indicates higher overall exposure to the contaminant family among individuals living in urban areas than in non-urban areas, whereas a negative β indicates lower exposure in urban areas. During pregnancy, living in urban areas corresponded to higher PC1 scores for persistent pesticides (*β* = 0.22; 95% CI 0.02, 0.44), phenols (*β* = 0.81; 95% CI 0.11, 1.53) and, less pronouncedly, for OP pesticide metabolites (*β* = 0.14; 95 CI −0.43, 0.68). Lower PC1 scores for phthalates were observed in urban participants (*β* = −0.30; 95% CI −0.73, 0.17), whereas PFAS levels showed little difference by urbanisation level (*β* = −0.04; 95% CI −0.28, 0.18).

In children, a relatively lower variance (37%) was accounted for by the first component of persistent pesticides compared to pregnant women. This component was primarily loaded of DDT (0.55) and PCB-170 (0.45); the first component of PFAS explained 56% of the total variance with loadings on PFUNDA (0.61), PFOS (0.48), PFHxS (0.44); the first phthalates’ component showed high loadings on sumDEPH (0.42), MEHP (0.40), MEOHP (0.40), MECPP (0.40), and MEHHP (0.39), explaining 50% of the total variance; the component of the phenol family explained 54% of the total variance and was characterised by loadings on PRPA (0.84) and MEPA (0.21); the first component of OP pesticide metabolites explained 46% of the total variance and was loaded of DEP (0.62), DETP (0.55) and DMTP (0.42).

Unlike for pregnant women, children living in urban areas tended to have lower PC1 scores for PFAS, and, less pronouncedly, for persistent pesticides and OP pesticide metabolite components (*β* = −0.23; 95% CI −0.45, −0.01; *β* = -0.18; 95% CI −0.52, 0.16 and *β* = −0.07; 95% CI −0.34, 0.22, respectively). Conversely, urban residence in children corresponded to higher PC1 scores for phthalates and phenols (β = 0.26; 95% CI −0.10, 0.63; *β* = 0.53; 95% CI −0.03, 1.12, respectively).

**Fig. 3:**
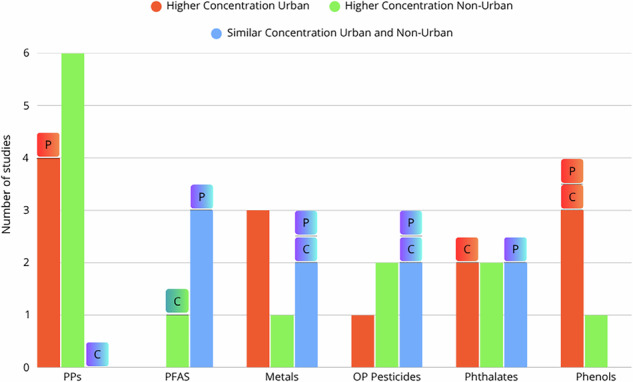
The y-axis represents the number of studies identified in the literature, comparing concentrations of contaminants across various families in urban and non-urban settings (x-axis). Studies are colour-coded: red indicates higher contaminant concentrations in urban areas, green indicates higher concentrations in non-urban areas, and blue indicates similar concentrations in both. Our study results are visually represented by the addition of half a study in the relative bar, labelled ‘P’ for results related to pregnancy and ‘C’ for children. PPs Persistent Pesticides, PFAS Per- and polyfluoroalkyl substances, OP Organophosphate, P Pregnancy, C Children.

Figure 3 summarises findings from thirty-five biomonitoring studies, including our own, comparing contaminant concentrations in urban and non-urban areas. It shows the number of studies we identified and examined (y-axis), comparing concentrations per family of contaminants in urban and non-urban settings (x-axis). Studies were classified by contaminant family (persistent pesticides, PFAS, metals, OP pesticides, phthalates, and phenols) and by whether concentrations were higher (red), lower (green), or similar (blue) in urban versus non-urban settings. The figure provides a visual overview of the existing literature, contextualising our study results within previously reported exposure patterns.

## Discussion

This study examined concentrations of a large number of environmental contaminants from six families, comparing distributions between urban and non-urban areas of residence for pregnant women and their children aged 6–11 years from five European countries. Our results showed varying patterns of exposure across levels of urbanisation and between life stages (pregnancy or childhood). During pregnancy, mothers living in urban areas showed higher concentrations of environmental contaminants than those living in non-urban areas, with a pronounced increase in phenols and persistent pesticides. In contrast, childhood exposure patterns showed more diversity among contaminant families, with children in urban areas having lower levels of PFASs and higher levels of phenols and phthalates compared to children in non-urban areas.

Regarding the correlation patterns between the two time periods, pregnancy and childhood, we generally observed low correlations overall. However, correlations were higher for persistent compounds, such as banned pesticides like DDT—likely due to their stability in the environment shared by mothers and children—and PFAS, whose strong C–F bonds confer chemical and thermal stability. No consistent patterns were observed for many other compounds, particularly phenols and OP pesticides, which is reasonable given their higher polarity, water solubility, and smaller molecular size.

The literature provides evidence of a complex picture and varied distribution patterns of contaminants across urban, rural, and industrial areas, along with associated health implications and burdens. We sought to assess where our results fit into the landscape provided by studies that we identified as the most relevant in the literature to address our research question. Although this is not intended to be a systematic literature review, it can offer a valid perspective, allowing visualisation and a better understanding of what is already known on this topic. We classified studies, including our, based on whether they found higher, lower or similar concentrations of environmental contaminants in the comparison between urban and non-urban areas. Overall, six studies found higher concentrations of persistent pesticides in non-urban areas, although in the other four studies, including our finding on the pregnancy period, the opposite was observed. Three studies found similar concentrations of PFAS (as with our pregnancy finding), with one exception [23] where higher concentrations were found in non-urban areas (similar to our results on children). The situation was more heterogeneous with metals, with three studies reporting higher concentrations in urban populations, consistent with our findings for arsenic and lead. In the literature on OP pesticides concentrations, the same number of studies found similar concentrations in urban and non-urban areas or higher in non-urban settings, and our study adds to those that found similar concentrations. Notably, we found that children living in non-urban regions had higher exposure to DETP, exceeding that of their urban counterparts by more than 20%. Three studies reported heterogeneous phthalate concentrations in both environmental settings, whereas our results suggest higher concentrations in the urban environment among pregnant women. However, for children, the concentration levels were more varied. Interestingly, most studies (four) reported higher concentrations of phenols in urban settings, a trend confirmed in our study in both pregnant women and children.

The primary sources of exposure to these compounds are associated with dietary habits and drinking water [24–26], with socio-economic status being a significant determinant of exposure [27]. An overview of where these compounds can be found, along with their chemical characteristics, is available in the Supplementary Material [28].

### Strength and limitations

The main strength of our study lies in its prospective design, involving 1021 mother-child pairs from five European countries, and in its investigation of two critical stages of a child’s life: foetal life and childhood (6-11 years old). Environmental exposures were estimated by measuring internal contaminant concentrations and biomarkers in biological media, which provide valuable information on exposure levels. Furthermore, our study adopts a multi-cohort approach, akin to similar studies, with several advantages, such as increased generalisability and enhanced confidence in the replicability of findings thanks to the heterogeneity between different populations [29]. Moreover, this study provides concentration levels of many contaminants across five different European countries, contributing to enriching the information gathered by biomonitoring studies, such as those included in the HBM4EU initiative [28]. However, understanding the possible causes of differences in the environmental contaminant concentrations between urban and non-urban areas constitutes a complex undertaking. Our study, which is fundamentally descriptive in nature, is limited to characterising the burden of these exposures in urban and non-urban areas in different regions within different European countries, without the ambition of establishing causal links.

Among the limitations of the study, it should be noted that differences between urban and non-urban areas can extend beyond settlement size and population density, as captured by the degree of urbanisation indicator used. Although practical, this indicator lacks more detailed information on geographical conformation or areas whose historical contamination as industrial sites, waste dumpsites or mining areas is known. We also acknowledge that collapsing the seven GHS-SMOD categories into two broad groups (“urban” vs. “non-urban”) may have reduced precision and increased the risk of exposure misclassification among participants, potentially affecting our analysis. In addition, our exposure measure, based on residential address, does not capture differences in individuals’ daytime environments, as is common in studies using residence-based exposure estimates.

Moreover, other factors likely acting as distal determinants of exposure, such as institutional and socio-economic infrastructures, cultural dynamics, ecosystem influences, and health-related policies, alongside historical, societal and ecological conditions, can largely contribute to exposure disparities across different degrees of urbanisation areas [30–32]. All this information could help improve the evaluation of how urban or non-urban areas may have influenced the distribution of environmental contaminants in our study population.

Additionally, the study faced constraints due to limited population size within each degree of urbanisation, coupled with missing values for some environmental contaminants. This resulted in a non-uniform distribution of subjects between urban and non-urban areas, requiring aggregation of the peri-urban and rural degrees into a unique group, and resulting in a loss of gradient information, especially for individuals residing in suburban areas.

Furthermore, potential biases and measurement errors were discussed in previous studies using chemical exposome data, particularly during urine and blood sample collection [33]. Despite efforts such as automation and reference measurement systems to mitigate these issues, common laboratory biases could still arise within laboratories. Moreover, the single-time sampling, for pregnant women and children, particularly for non-persistent chemicals, such as OP pesticides and metals, does not capture temporal variability in exposures [34].

Overall, these findings provide a useful overview of differences in the burden of exposure to environmental contaminants between urban and non-urban areas in both pregnancy and childhood, although with some differences across the two life stages. Results are mostly homogenous among cohorts, with the exception of PCBs. Our study can be seen as an initial step towards generating hypotheses about and, subsequently, understanding the mechanisms that generate unequal distributions of exposure loads on certain population subgroups.

## Supplementary information

Supplementary Information

## Data Availability

If you wish to obtain HELIX data, please follow the HELIX External Data Request Procedures, starting with the submission of a request form provided to helixdata@isglobal.org. Detailed codebooks can be made available on request by sending an email to the HELIX Data Manager jose.urquiza@isglobal.org.

## References

[1] Cohen Hubal EA, Reif DM, Slover R, Mullikin A, Little JC. Children’s environmental health: a systems approach for anticipating impacts from chemicals. Int J Environ Res Public Health. 2020;17:8337. 33187264 10.3390/ijerph17228337 PMC7696947

[2] Adeola FO. Global Impact of Chemicals and Toxic Substances on Human Health and the Environment. In: Haring R, Kickbusch I, Ganten D, Moeti M, editors. Handbook of Global Health. Springer International Publishing; 2021:1–30.

[3] Sly PD, Flack F. Susceptibility of children to environmental pollutants. Ann th e N Y Acad Sci. 2008;1140:163–83. 10.1196/annals.1454.017 18991915

[4] Pascale A, Laborde A. Impact of pesticide exposure in childhood. Rev Environ Health. 2020;35:221–7. 32598326 10.1515/reveh-2020-0011

[5] Vrijheid M, Fossati S, Maitre L, Marquez S, Roumeliotaki T, Agier L, et al. Early-life environmental exposures and childhood obesity: an exposome-wide approach. Environ Health Perspect. 2020;128:67009. 32579081 10.1289/EHP5975 PMC7313401

[6] Goldman LR, Koduru S. Chemicals in the environment and developmental toxicity to children: a public health and policy perspective. Environ Health Perspect. 2000;108:443–8. 10852843 10.1289/ehp.00108s3443 PMC1637825

[7] EUROSTAT. Chemicals production and consumption statistics 2025 [Available from: https://ec.europa.eu/eurostat/statistics-explained/index.php?title=Chemicals_production_and_consumption_statistics.

[8] Özel F, Rüegg J. Exposure to endocrine-disrupting chemicals and implications for neurodevelopment. Develop Med Child Neurol. 2023;65:1005–11. 36808586 10.1111/dmcn.15551

[9] Evans GS, Cadogan D, Flueckiger A, Hennes C, Kimber I. Chemical pollution, respiratory allergy and asthma: a perspective. J Appl Toxicol. 2008;28:1–5. 17726695 10.1002/jat.1294

[10] Legler J, Fletcher T, Govarts E, Porta M, Blumberg B, Heindel JJ, et al. Obesity, diabetes, and associated costs of exposure to endocrine-disrupting chemicals in the European Union. J Clin Endocrinol Metab. 2015;100:1278–88. 25742518 10.1210/jc.2014-4326 PMC4399302

[11] European Commission aSOotEU. Applying the Degree of Urbanisation - A methodological manual to define cities, towns and rural areas for international comparisons. European Commission, 2021.

[12] Maitre L, de Bont J, Casas M, Robinson O, Aasvang GM, Agier L, et al. Human early life exposome (HELIX) study: a European population-based exposome cohort. BMJ Open. 2018;8:e021311. 30206078 10.1136/bmjopen-2017-021311 PMC6144482

[13] Wright J, Small N, Raynor P, Tuffnell D, Bhopal R, Cameron N, et al. Cohort profile: the born in Bradford multi-ethnic family cohort study. Int J Epidemiol. 2013;42:978–91. 23064411 10.1093/ije/dys112

[14] Heude B, Forhan A, Slama R, Douhaud L, Bedel S, Saurel-Cubizolles M-J, et al. Cohort profile: the EDEN mother-child cohort on the prenatal and early postnatal determinants of child health and development. Int J Epidemiol. 2016;45:353–63. 26283636 10.1093/ije/dyv151

[15] Guxens M, Ballester F, Espada M, Fernández MF, Grimalt JO, Ibarluzea J, et al. Cohort profile: the INMA—INfancia y Medio Ambiente—(Environment and Childhood) project. Int J Epidemiol. 2012;41:930–40. 21471022 10.1093/ije/dyr054

[16] Grazuleviciene R, Danileviciute A, Nadisauskiene R, Vencloviene J. Maternal smoking, GSTM1 and GSTT1 polymorphism and susceptibility to adverse pregnancy outcomes. Int J Environ Res Public Health. 2009;6:1282–97. 19440446 10.3390/ijerph6031282 PMC2672398

[17] Chatzi L, Leventakou V, Vafeiadi M, Koutra K, Roumeliotaki T, Chalkiadaki G, et al. Cohort profile: the mother-child cohort in Crete, Greece (Rhea Study). Int J Epidemiol. 2017;46:1392–3k. 29040580 10.1093/ije/dyx084

[18] Florczyk AJ. GHSL data package 2019. Luxembourg; 2019.

[19] GHS-SMOD R2019A - GHS settlement layers, updated and refined REGIO model 2014 in application to GHS-BUILT R2018A and GHS-POP R2019A, multitemporal (1975-1990-2000-2015) [Internet]. European Commission, Joint Research Centre. 2019.

[20] Haug LS, Sakhi AK, Cequier E, Casas M, Maitre L, Basagana X, et al. In-utero and childhood chemical exposome in six European mother-child cohorts. Environ Int. 2018;121:751–63. 30326459 10.1016/j.envint.2018.09.056

[21] Liljequist D, Elfving B, Skavberg Roaldsen K. Intraclass correlation – A discussion and demonstration of basic features. PLoS ONE. 2019;14:e0219854. 31329615 10.1371/journal.pone.0219854 PMC6645485

[22] D’Archivio M, Coppola L, Masella R, Tammaro A, La Rocca C. Sex and gender differences on the impact of metabolism-disrupting chemicals on obesity: a systematic review. Nutrients. 2024;16:181. 10.3390/nu16020181 PMC10818535 38257074

[23] DeLuca NM, Thomas K, Mullikin A, Slover R, Stanek LW, Pilant AN, et al. Geographic and demographic variability in serum PFAS concentrations for pregnant women in the United States. J Exposure Sci Environ Epidemiol. 2023;33:710–24. 10.1038/s41370-023-00520-6 PMC10541323 36697764

[24] Gkika IS, Arie Vonk J, Ter Laak TL, van Gestel CAM, Dijkstra J, Groffen T, et al. Strong bioaccumulation of a wide variety of PFAS in a contaminated terrestrial and aquatic ecosystem. Environ Int. 2025;202:109629. 40578111 10.1016/j.envint.2025.109629

[25] Morck TA, Erdmann SE, Long M, Mathiesen L, Nielsen F, Siersma VD, et al. PCB concentrations and dioxin-like activity in blood samples from Danish school children and their mothers living in urban and rural areas. Basic Clin Pharm Toxicol. 2014;115:134–44. 10.1111/bcpt.12214 24528479

[26] Muncke J, Andersson A-M, Backhaus T, Boucher JM, Carney Almroth B, Castillo Castillo A, et al. Impacts of food contact chemicals on human health: a consensus statement. Environ Health. 2020;19:s12940. 10.1186/s12940-020-0572-5 PMC7053054 32122363

[27] Montazeri P, Thomsen C, Casas M, de Bont J, Haug LS, Maitre L, et al. Socioeconomic position and exposure to multiple environmental chemical contaminants in six European mother-child cohorts. Int J Hyg Environ Health. 2019;222:864–72. 31010791 10.1016/j.ijheh.2019.04.002 PMC8713641

[28] Vorkamp K, Esteban Lopez M, Gilles L, Goen T, Govarts E, Hajeb P, et al. Coordination of chemical analyses under the European Human Biomonitoring Initiative (HBM4EU): concepts, procedures and lessons learnt. Int J Hyg Environ Health. 2023;251:114183. 37148759 10.1016/j.ijheh.2023.114183

[29] O’Connor M, Spry E, Patton G, Moreno-Betancur M, Arnup S, Downes M, et al. Better together: advancing life course research through multi-cohort analytic approaches. Adv Life Course Res. 2022;53:100499. 36652217 10.1016/j.alcr.2022.100499

[30] Krieger N. Proximal, distal, and the politics of causation: what’s level got to do with it? Am J Public Health. 2008;98:221–30. 18172144 10.2105/AJPH.2007.111278 PMC2376874

[31] Krieger N, Krieger N. Praise for Ecosocial Theory, Embodied Truths, and the People’s Health. Ecosocial Theory, Embodied Truths, and the People’s Health. 1 ed: Oxford University Press; 2021:i-iii.

[32] Pasqualini M. Subjective well-being in rural and urban areas under the COVID-19 crisis in France. In: Johansen PH, Tietjen A, Iversen EB, Lolle HL, Fisker JK, editors. Rural quality of life: Manchester University Press:2023.

[33] Agier L, Basagaña X, Maitre L, Granum B, Bird PK, Casas M, et al. Early-life exposome and lung function in children in Europe: an analysis of data from the longitudinal, population-based HELIX cohort. Lancet Planet Health. 2019;3:e81–e92. 30737192 10.1016/S2542-5196(19)30010-5

[34] Theodorsson E, Magnusson B, Leito I. Bias in clinical chemistry. Bioanalysis. 2014;6:2855–75. 25486232 10.4155/bio.14.249

