# Supplementary material for: Chemical exposome patterns in mothers and children across urbanisation levels in five European birth cohorts
Source: J Expo Sci Environ Epidemiol. 2026 Apr 2;36(4):619–28. doi: 10.1038/s41370-026-00859-6 (PMC13331734; doi:10.1038/s41370-026-00859-6)
Supplement: Supplementary file 1 — Supplementary Information [file 41370_2026_859_MOESM1_ESM.docx]

| **­­­­Cohorts** | **Non-urban area**  **(n)** | **Urban Area**  **(n)** | **Total**  **(n)** |
| --- | --- | --- | --- |
| **BiB** | 9 | 196 | 205 |
| **EDEN** | 175 | 23 | 198 |
| **KANC** | 27 | 169 | 196 |
| **RHEA** | 79 | 120 | 199 |
| **INMA-SAB** | 17 | 206 | 223 |
| **Total** | 307 | 714 | 1021 |

Table 1S. shows the distribution of mother-child pairs between non-urban and urban areas across five different birth cohorts: BiB (Born in Bradford, UK), EDEN (Etude des Déterminants de l’Enfant pré et postnatals du développement et de la santé, France), KANC (Kaunas cohort, Lithuania), RHEA (Mother-Child Cohort study, Crete, Greece), and INMA-SAB (INfancia y Medio Ambiente cohort, Spain).


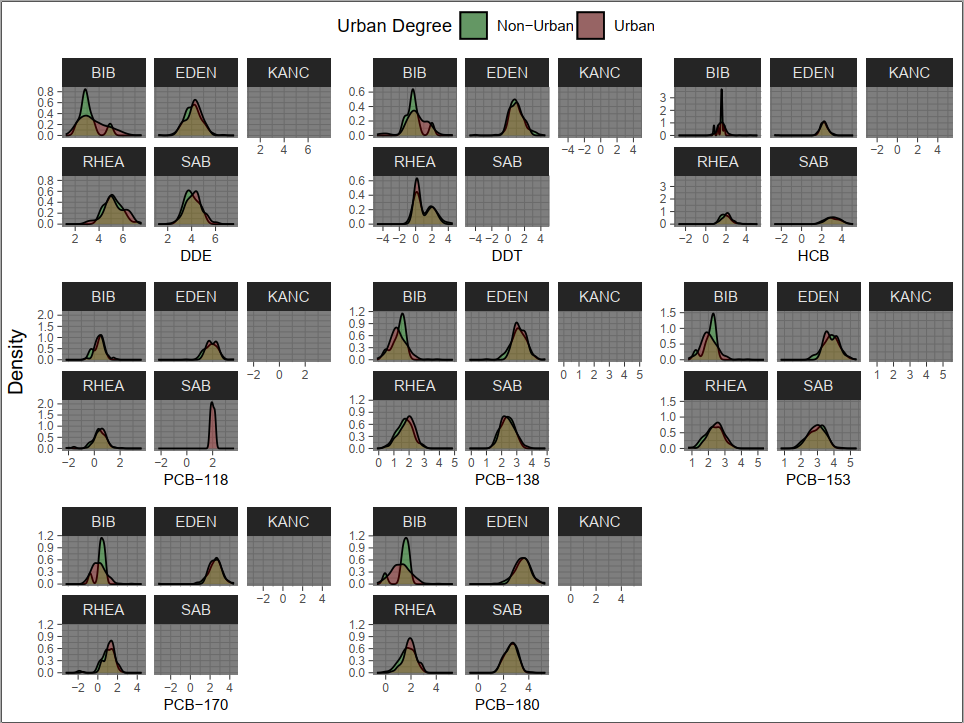


Fig S2. Probability density functions of compounds within POPs family focuses on comparing their concentration distribution during pregnancy between urban and non-urban areas.


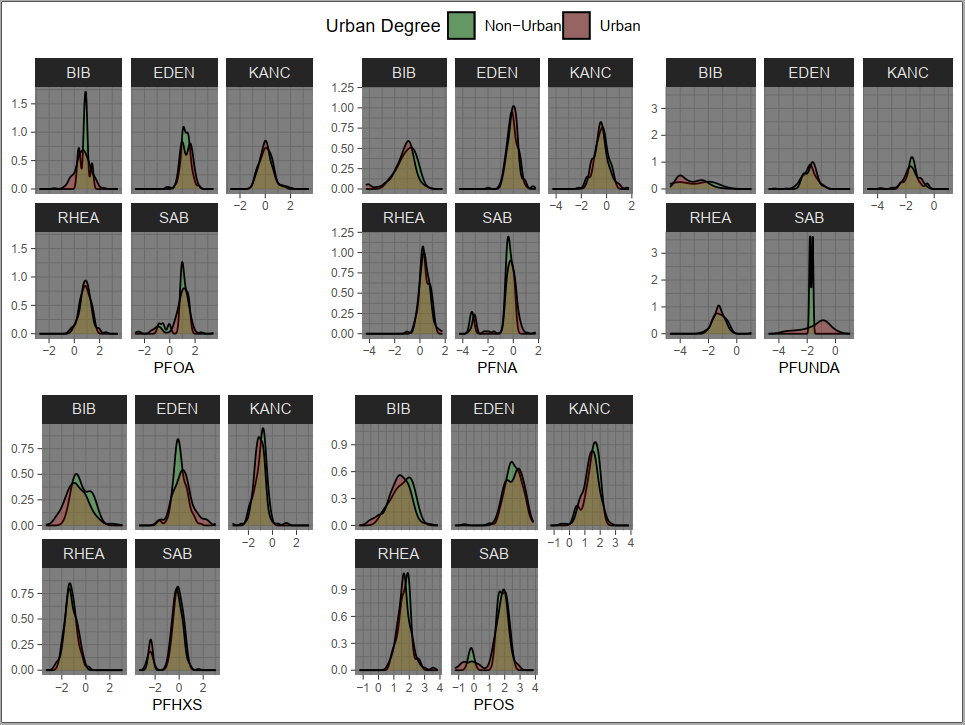


Fig S3. Probability density functions of compounds within PFASs family focuses on comparing their concentration distribution during pregnancy between urban and non-urban areas.


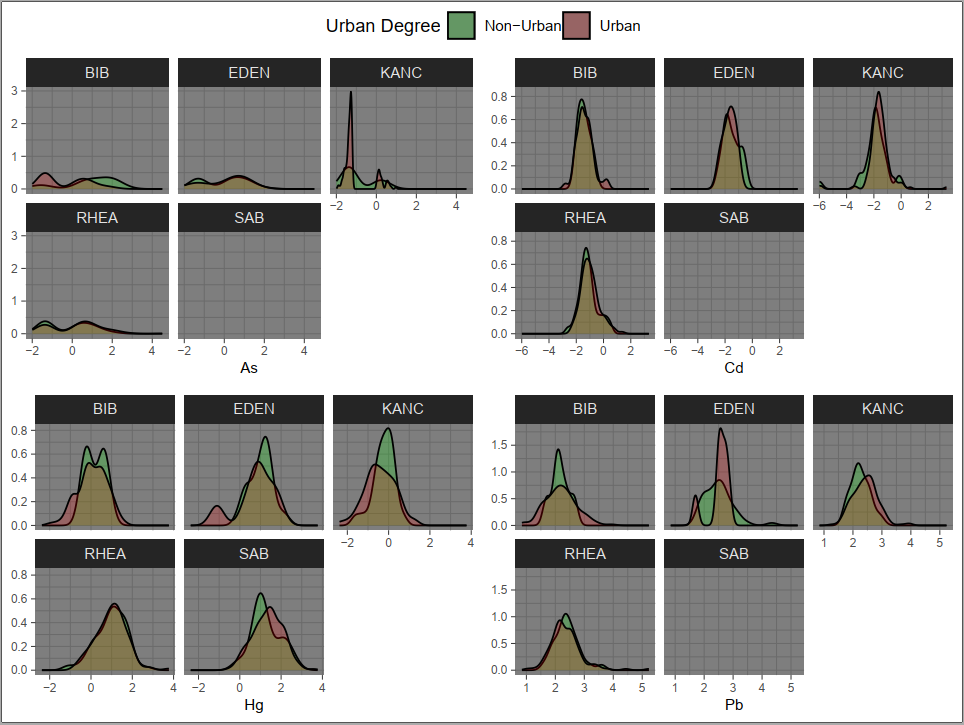


Fig. S4. Probability density functions of compounds within metals family focuses on comparing their concentration distribution during pregnancy between urban and non-urban areas.


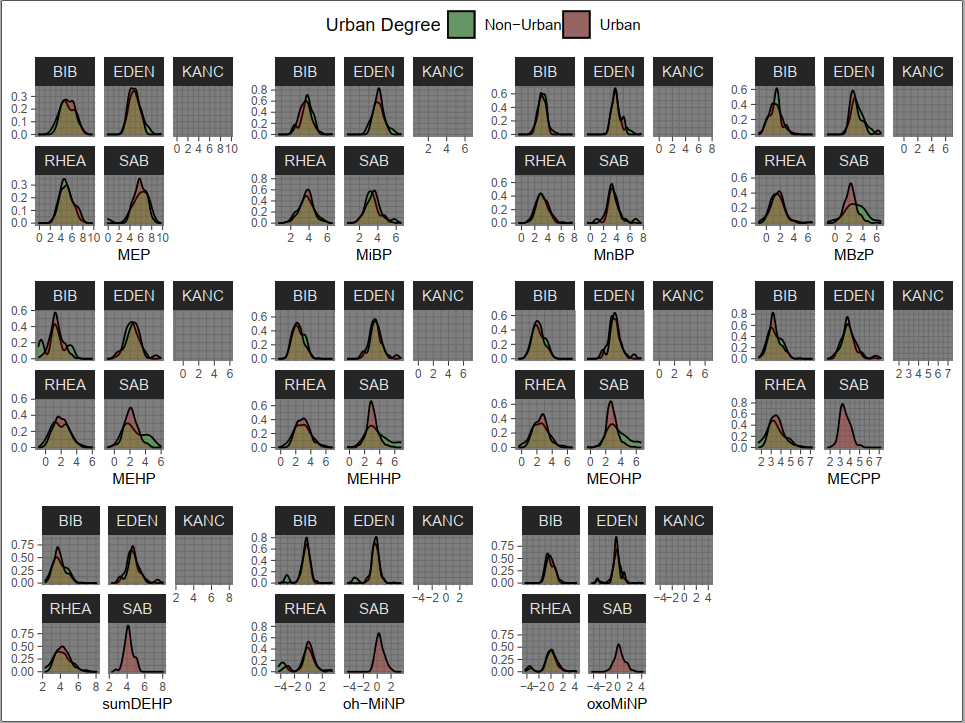


Fig 5S. Probability density functions of compounds within phthalates family focuses on comparing their concentration distribution during pregnancy between urban and non-urban areas.


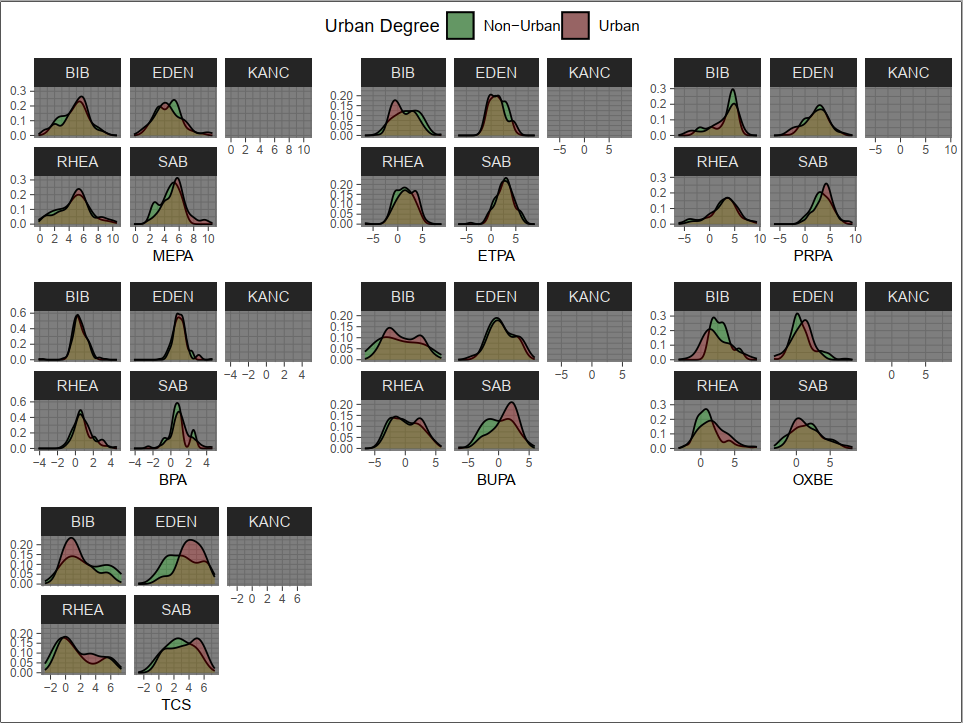


Fig S6. Probability density functions of compounds within phenols family focuses on comparing their concentration distribution during pregnancy between urban and non-urban areas.


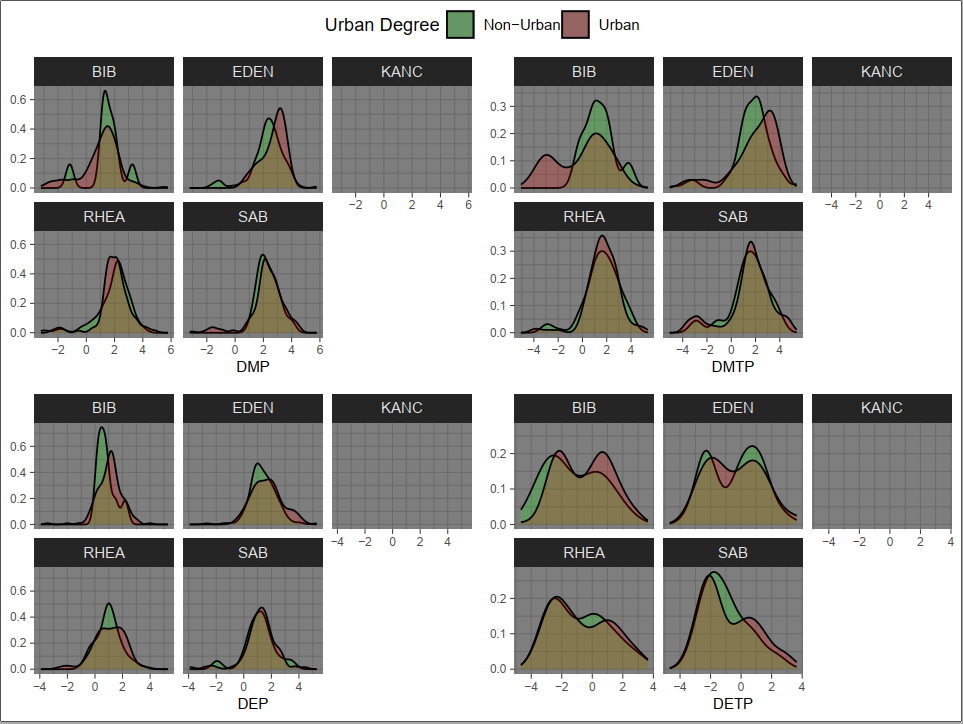


Fig. 7S. Probability density functions of compounds within OP pesticides family focuses on comparing their concentration distribution during pregnancy between urban and non-urban areas.


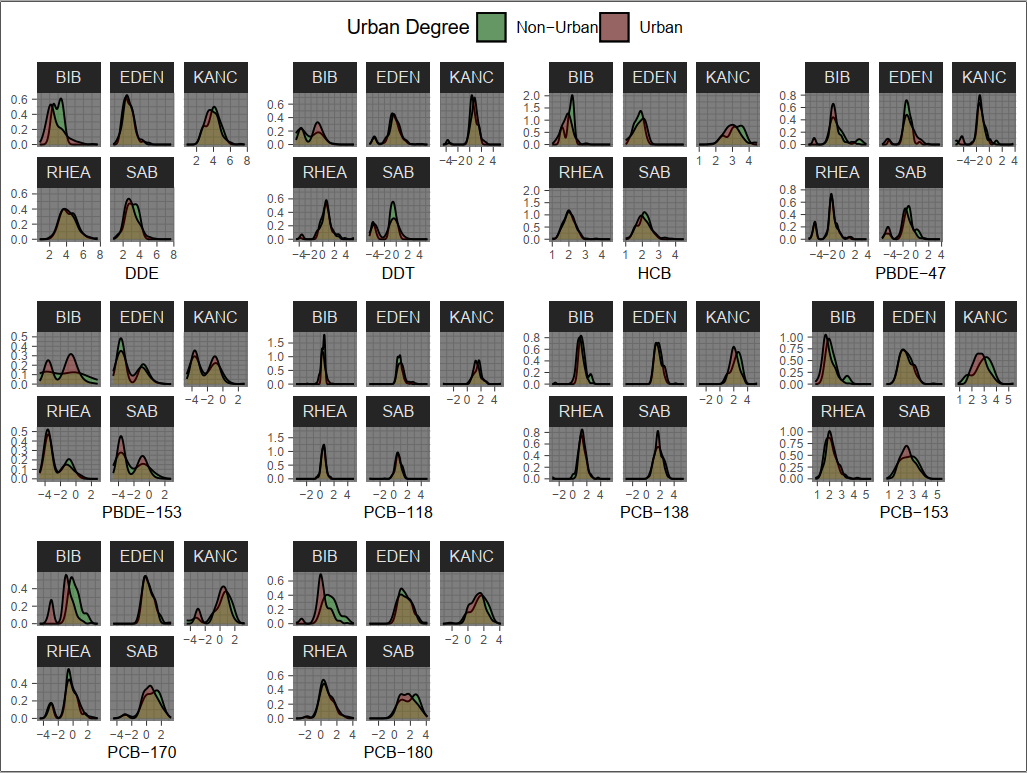


Fig 8S. Probability density functions of compounds within POPs family focuses on comparing their concentration distribution during childhood between urban and non-urban areas.


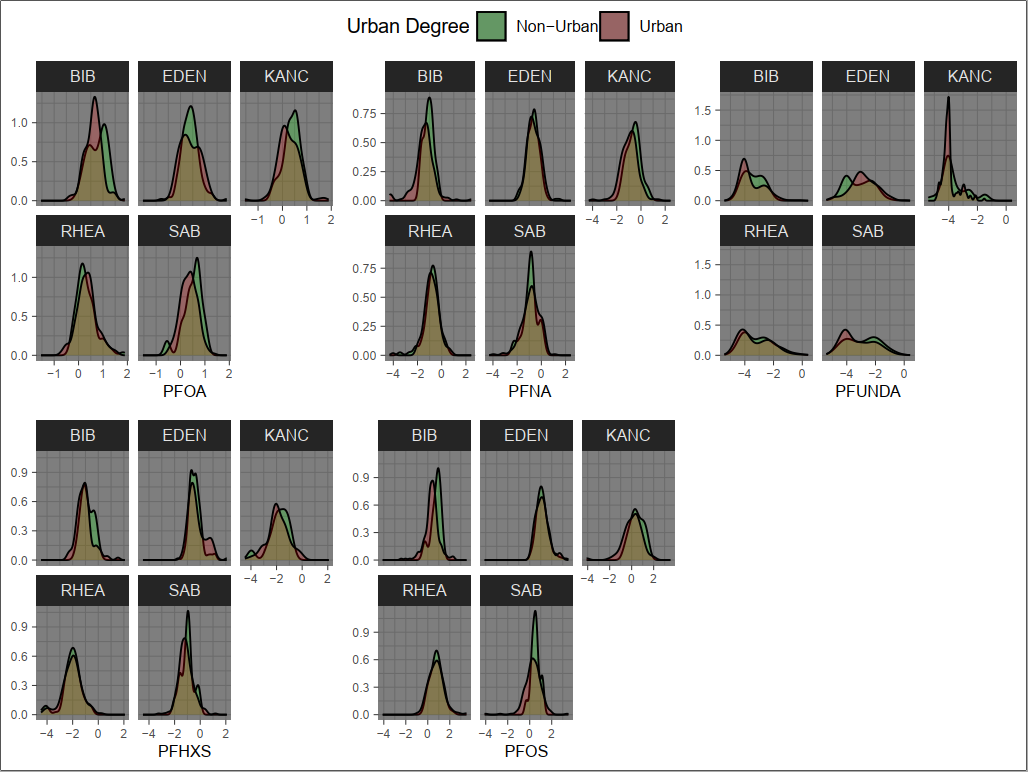


Fig 9S. Probability density functions of compounds within PFASs family focuses on comparing their concentration distribution during childhood between urban and non-urban areas.


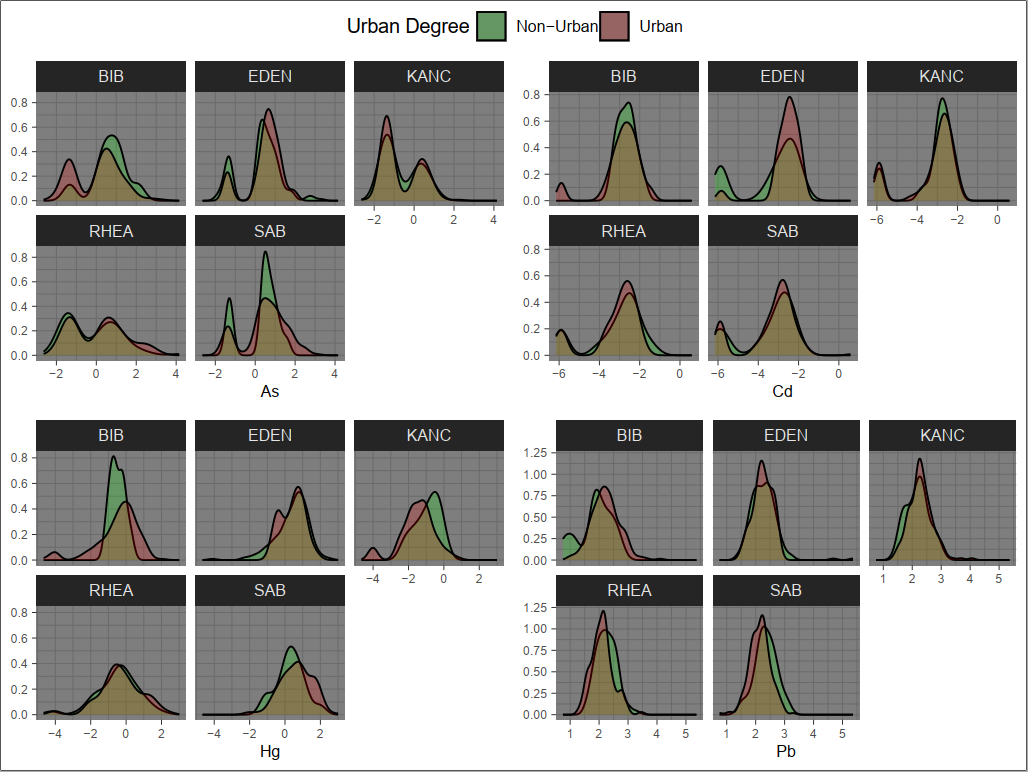


Fig 10S. Probability density functions of compounds within metals family focuses on comparing their concentration distribution during childhood between urban and non-urban areas.


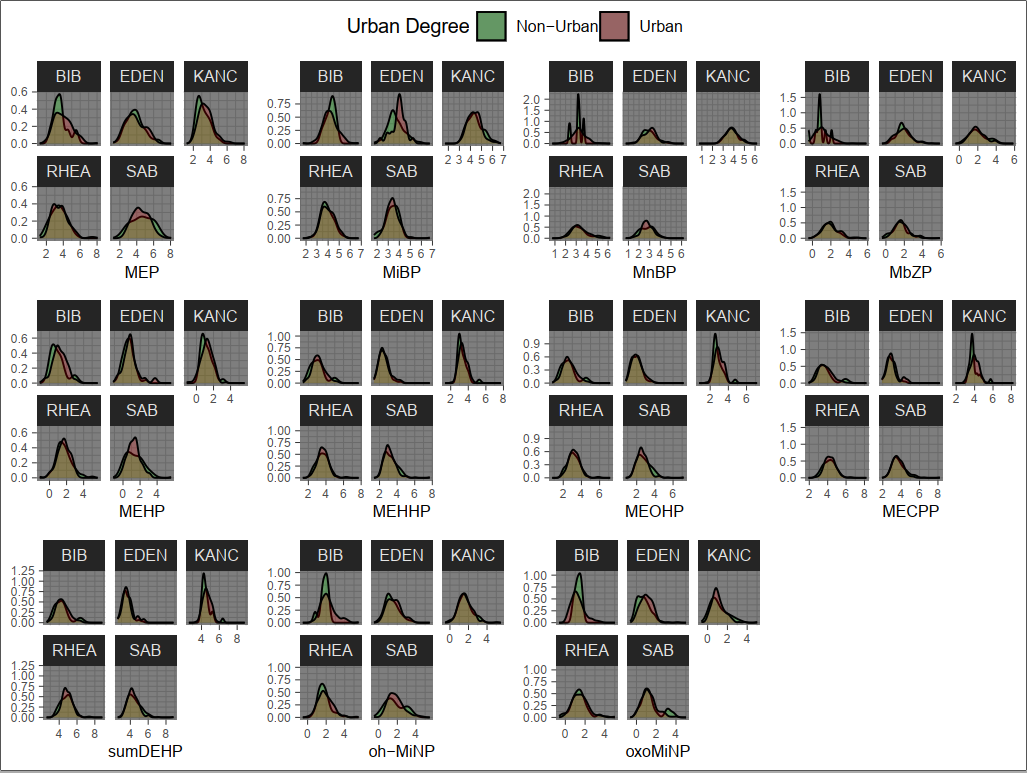


Fig 11S. Probability density functions of compounds within phthalates family focuses on comparing their concentration distribution during childhood between urban and non-urban areas.


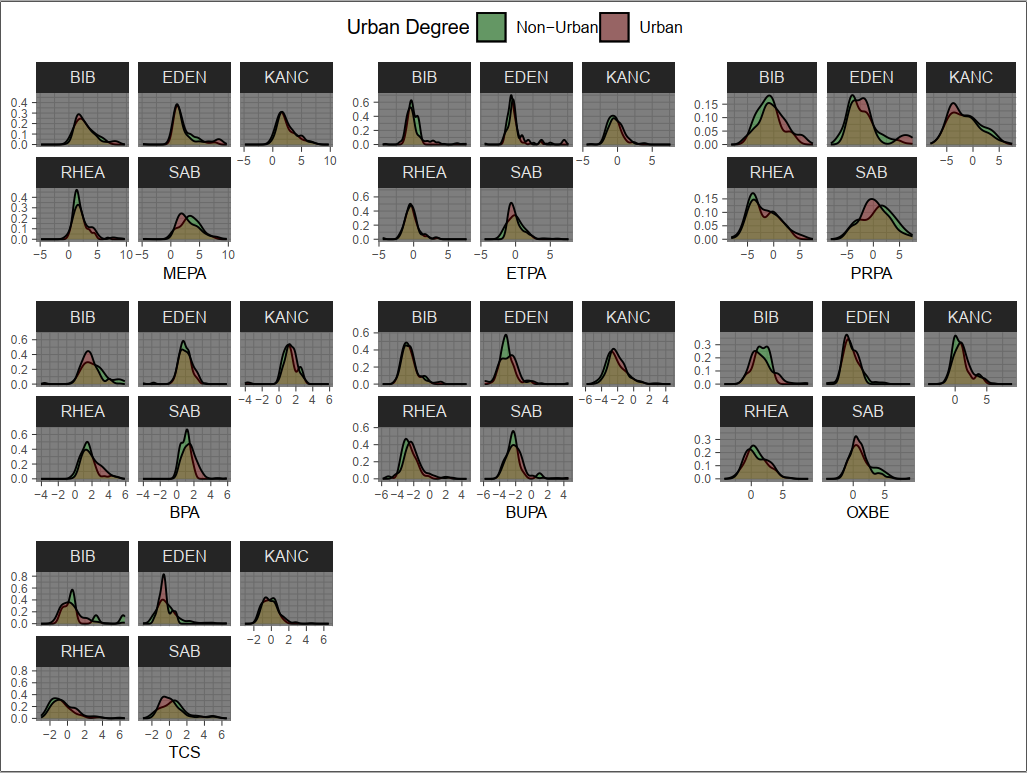


Fig 12S. Probability density functions of compounds within phenols family focuses on comparing their concentration distribution during childhood between urban and non-urban areas.


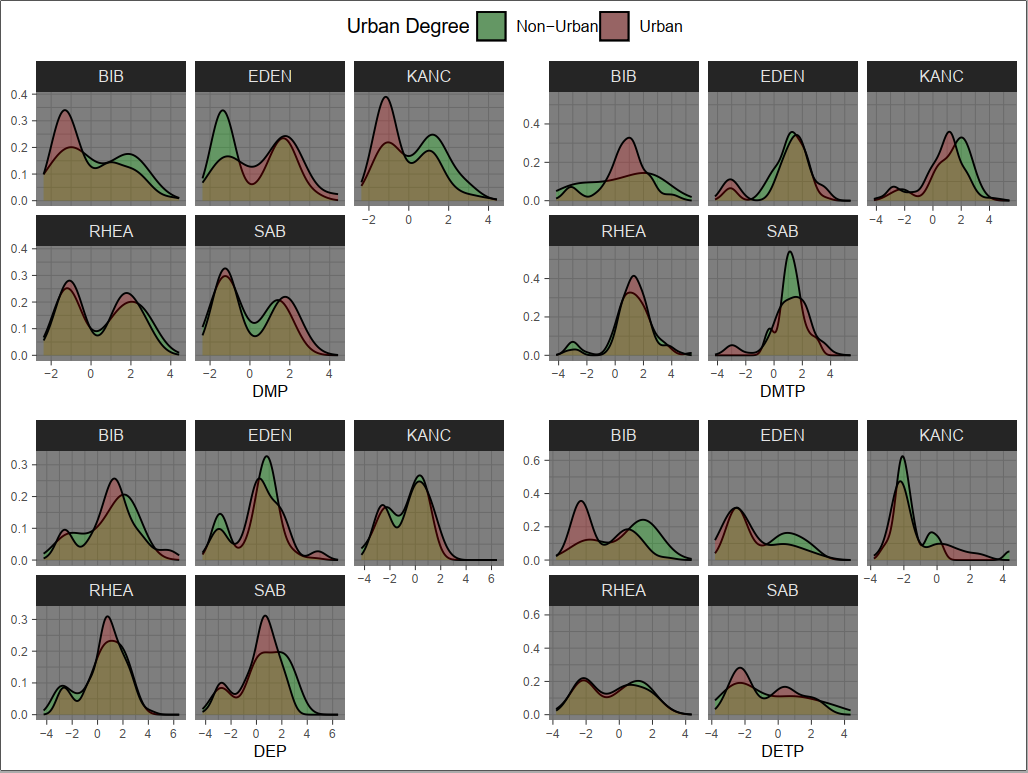


Fig 13S. Probability density functions of compounds within OP pesticides family focuses on comparing their concentration distribution during childhood between urban and non-urban areas.

**Chemical Description and Possible Sources of Exposure:**

***Persistent organic pollutants (DDE, DDT, HCB, PCBs, PBDEs)***

DDT and HCB substances were commonly used in the 60-70s as fungicides while PCBs and PBDEs are used as plasticizers, sealants and inks. A significant amount of these compounds can be released from fires or incinerators, and their distribution can be facilitated by atmospheric transport, human activities, and migratory birds, even over long distances (Jin et al., 2008). These halogenated compounds are particularly hazardous because they exhibit slow biodegradation in the environment due to their lipophilic nature (Guang-Zuh et al., 2007), tending to bioaccumulate and biomagnify (Kodavanti et al., 2014). For instance, DDT has been reported to persist in the body, particularly in adipose tissues and lipid-rich human tissues (e.g., blood and muscles), for up to 50 years (Mrema et al., 2013). Adult exposure to these contaminants is primarily linked to dietary sources with early exposure often occurring through placental transfer and during breastfeeding to the foetus (Perera et al., 2005).

***Similar to our results***

Morck et al., 2014 conducted a study in Denmark examining plasma levels of 143 mothers and 116 children (aged 6-11 years). Mothers and children residing in non-urban areas exhibited significantly lower levels of PCBs, DDT, and DDE between mothers. These differences in concentration were attributed to distinct lifestyles and urban environments compared to those residing in rural areas.

Knudsen et al., 2016, similar to our findings, in a study conducted in Denmark, it was found that PBDEs concentration levels were higher among children in rural areas, possibly due to older house construction with higher PCB-28 concentration levels, associated with furniture such as carpets and sealing materials

***Different to our results***

Elvia et al., 2000 conducted a study in Mexico demonstrated that DDT metabolite levels were five times higher in maternal milk samples of women living in rural areas compared to urban areas. This suggests that differences in exposure levels may be attributed to occupational exposures or the consumption of animal-derived food products.

Cruz et al., 2005 evaluated organochlorine pesticides among urban and rural adult populations in Portugal showing lower levels of DDT and HCB among rural residents. This phenomenon was attributed to the consumption of imported food products from Latin America and Africa, where DDT was still in use. Urban residents tend to consume more imported goods compared to their rural counterparts, who typically favour locally sourced products.

Wielgomas et al., 2013 and Wang et al., 2015 investigated children exposure to persistent pesticides in urban and rural populations in Poland and China was higher among children residing in rural areas compared to those in urban environments.

Arcury et al., 2021, different from our results, a community-based research study conducted in North Carolina compared agricultural and non-agricultural communities, revealing that non-urban-dwelling children had lower concentrations of organochlorine and pyrethroid pesticides.

***PFASs***

Differences in PFAS concentration levels can be the result of exposure from different sources. PFAS compounds show near-ubiquity in the environment, characterised by high thermal and chemical stability. They have a propensity to bioaccumulate and biomagnify in food chains, are present in air, water, and soil, and emissions are primarily originated from industrial and municipal waste related to the production of fluoropolymers, building construction, food packaging, textiles, paints, inks, and medical devices (Gluge et al.,2020). Also, they manifest multiple routes of exposure and can be detected in various matrices, including blood, milk, urine, organs, and tissues, and associated with carcinogenic, immunotoxic, hepatotoxic, and reprotoxic activities (Panieri et al., 2022). However, comparative literature on PFAS compound concentrations in urban and non-urban areas is limited despite that geographic location was found to be a strong predictor of PFAS concentrations. For example, sources of PFAS exposures measured in pregnant women across different counties in the US, with different urban and non-urban gradients, were mainly attributed to drinking water and air contamination. The latter was attributed to the use of firefighting foam in nearby military installations and airports, and contaminants spill from manufacturing facilities (DeLuca et al., 2023).

***Similar to our results***

Different studies (Sasaki et al., 2003; Harada et al., 2005; Harada et al., 2006; Fromme et al., 2009) in Japan indicated fluctuations in the mean concentrations of PFOS and PFOA in ambient air between rural and urban areas, depending on the sampling year. Although the concentrations of PFOS and PFOA in these studies are notably lower than the levels typically observed in human samples, they emphasise the variability in exposure levels between urban and non-urban areas, also through inhalation.

Morck et al., 2015 showed relatively similar results, in a study investigating six measured PFAS concentrations in plasma samples obtained from over 100 Danish school children, reported no significant differences between urban and rural areas. However, high concentrations of PFOS were detected in mothers.

Runkel et al., 2023 carried out a study in three agricultural areas in northeastern Slovenia revealed that children in regions with poor water quality and specific geographical and diet factors, such as reduced local winds due to the Alpine barrier and high seafood consumption, had elevated PFAS concentrations.

***Metals (As, Cd, Hg, Pb)***

Identifying pathways of human exposure to metals is challenging due to the wide range of sources in the environment. Arsenic in gaseous form and inorganic and organic salts can be mainly found in drinking water, pesticides, food chain, defoliants, and electric devices (Kuivenhoven et al., 2019), while cadmium and related compounds are prevalent in food and dietary supplements, paint pigments, PVC materials, and tobacco smoke (Friberg et al., 2018, Elinder et al., 2019). Mercury and its inorganic and organic compounds are often associated with cosmetics, fish consumption, gold mining areas (Yang et al., 2020), while lead is found in plumbing, plastics, food, toys, paints, and gasoline (Nieboer et al., 2013). Notably, all of these metals and their compounds are present in tobacco cigarettes, albeit in varying concentrations. Lead and cadmium, in particular, are often the most abundant metals in tobacco cigarettes. In addition, the widespread industrial activities in Europe are among the major contributors to the contamination of air, water, and soil with metals (Caruso et al., 2014; Hunova et al., 2022).

***Similar to our results***

Al-Rajhi et al., 1996 conducted a study in Saudi Arabia, it was found that indoor and outdoor dust concentrations of cadmium were lower in rural areas compared to urban areas, corroborating the results of our study. This difference was attributed to traffic and industrial emissions. Additionally, lead concentrations were also observed to be lower in rural areas.

Laamech et al., 2014 showed similar to our results, no significant differences were found among children residing in urban and rural areas in Morocco regarding lead exposure, while mercury exposure levels were lower among children in rural areas compared to urban areas in both males and females. Interestingly, within rural areas, there was a gender difference in heavy metal exposure, with males exhibiting higher cadmium concentration levels, possibly due to behavioural differences.

Hruba et al. (2023) conducted a cross-sectional study across six European countries to investigate children's exposure to lead and mercury in "hot spots," urban, and rural areas. Concentration levels of these metals in hot spots were consistently higher across all countries compared to urban and rural areas. The prevalence of metallurgic industries in Eastern Europe during the 1950s-1960s, as demonstrated in Poland, may have contributed to these findings. Nevertheless, there was no consistent pattern of urban lead exposure linked to industrial expansion among the European countries evaluated. In line with our findings, minor differences in lead concentration levels were observed between urban and rural areas, but higher concentrations were noted among subjects living in rural areas in Poland, possibly associated with drinking water contamination or proximity to industrial emissions.

***Different to our results***

Aelion et al., 2019 showed blood lead levels in children aged <1 to 6 years in South Carolina resulted to be lower in rural areas compared to those living in urban areas, particularly in the age range of <1 year to 2 years.

Li et al., 2021 In contrast to our findings, a study examining more than 40 years of raw metal concentrations in China showed that the sample-weighted concentration of cadmium and lead in household dust in rural areas was 3.29 times higher than in urban areas, attributed to coal-fired cooking or the use of pesticides and fertilisers.

***Phthalate metabolites (MEP, MiBP, MnBP, MBzP, MEHP, MEHHP, MEOHP, MECPP, oh-MiNP, oxo-MiNP)***

Phthalates are recognised as endocrine-disrupting chemicals, capable of impacting various body systems, including metabolism, immunity, and reproduction (Ventrice et al., 2013). Phthalates are extensively employed as plasticizers in polyvinyl chloride (PVC), which finds applications in plastic packaging, hair and personal care products, pharmaceuticals, and medical devices (Wang et al., 2021).

***Similar to our results***

Carli et al., 2022 in the Italian LIFE PERSUADED biomonitoring project, no significant differences in DEHP metabolite levels were found between mothers living in urban and rural areas. MEHP and DEHP metabolite concentration levels were lower in urban areas in southern Italy but higher in urban areas in the centre of Italy, respectively. Interestingly, women living in southern Italy showed a higher conversion rate, indicating faster excretion, for DEHP compounds compared to those in the north and centre of Italy, while the conversion rate remained similar between urban and rural areas.

***Different to our results***

Frederiksen et al., 2013 conducted a study in Denmark collected urine samples of phthalate metabolites from children aged 6-11 and their respective mothers, revealing that children were more exposed to some phthalates than their mothers. However, no differences were found between urban and rural areas.

Cutanda et al., 2014 In the DEMOCOPHES project conducted in Spain showed that 5-OH-MiBP, the sum of DEHP metabolites, and MnBP urinary concentrations were significantly lower among children in urban areas compared to those in rural areas, and MEP exposure levels were lower in both mothers and children in urban areas, contrasting our results. Furthermore, there was considerable variability in exposure within the same location, especially in urban areas, influenced by the different use of personal care products, insecticidal and mosquito sprays.

Larsson et al., 2014 found that urinary concentration levels of low molecular weight phthalates, such as MBzP and MnBP, were lower among mothers and children living in rural areas compared to urban areas in Sweden. The authors attributed these differences to the home environment and the greater presence of PVC in floorings and wall coverings in rural areas.

Runkel et al., 2020 similarly in Slovenia, MEP, MEHP, MBzP, and MEHP concentration levels in children, as well as DEHP metabolites and MEP concentration levels in mothers, were significantly lower in urban areas compared to rural areas, with only MnBP concentration levels being higher in urban areas for both mothers and children, consistent with our findings for MnBP.

***Phenols (MEPA, ETPA, PRPA, BPA, BUPA, OXBE, TCS)***

Identifying specific routes of exposure for phenolic compounds is a complex task. Parabens, including methyl, ethyl, propyl, and butyl parabens, are commonly used as preservatives in a wide range of products, including food, beverages, cosmetics, and medicines. The Consumer Product Information Database highlights their presence in pet and personal care products, as well as toilet cleaners and air fresheners. BPA, along with parabens, has been widely used in the production of plastics for more than five decades. Although it was banned in Europe for toys and other products such as baby water bottles, pens, and markers in 2014, it continues to be used in food packaging and beverage cans. BPA can also be largely found in construction and building materials, such as flooring, tiles, bathtubs, sinks, insulation, and wall materials, as well as in furniture.

***Similar to our results***

Rakkestad, 2007; Rudel and Perovich, 2009; Runkel et al., 2022 analysed urinary concentration levels of three types of environmental phenols (bisphenols, parabens, and triclosan) in men and lactating primiparous women in Slovenia, which, individuals in urban and industrialised areas exhibited higher overall exposure levels compared to those in rural locations. The authors suggested that these differences might be primarily attributed to urban air exposure.

Frederiksen et al., 2013 showed that Danish mothers and their children in urban areas displayed higher concentration levels of benzophenone-3 and parabens than their rural counterparts. However, BPA concentration levels were marginally lower among both children and mothers in urban areas compared to those in rural areas. However, Carli et al., 2022 showed contrasting results on BPA. Women residing in urban areas showed higher BPA concentration compared to rural areas as well as in northern Italy compared to those living in the centre and south.

Larsson et al., 2014 conducted a study among Swedish mothers and their children revealed higher concentrations of parabens (such as methyl parabens and propyl parabens) in children from urban areas compared to those in rural regions.

***OP pesticide metabolites (DMP, DMTP, DEP, DETP)***

Exposure to organophosphate pesticide metabolites primarily occurs through food ingestion. Detecting these organophosphate metabolites is challenging due to their short half-life, with the majority being excreted in urine within 24 hours (Foxenberg et al., 2011). This complicates the comparison of exposure between different environmental settings.

***Similar to our results***

Bradman et al., 2015 explored the impact of diet on pesticide exposure in forty young children from both urban (Oakland) and agricultural (Salinas) areas in the United States. It was found that six organophosphate pesticide metabolites had higher concentrations among Salinas children, with adjusted concentrations of DMP (dimethyl phosphate) at GM = 97.7 ng/ml for Salinas children versus GM = 66.9 ng/ml for Oakland children.

Arcury et al., 2018 compared rural farmworker and urban non-farmworker children, found that urinary concentrations of chlorpyrifos metabolite, a commonly used organophosphate pesticide, were equivalent between the two groups, with a geometric mean of approximately 16 ng g^-1^. This pattern was confirmed by a study involving women as well. However, rural farmworker children displayed higher detection rates of the molecule. This observation underscores the widespread nature of pesticide exposure in humans, extending beyond specific activities like agriculture or industries where pesticides are extensively used or produced. It emphasises the need for awareness regarding the potential exposure to these compounds, especially among children at risk of neurocognitive conditions (Arcury et al., 2021).

Gonzalez et al. (2020) reported that children aged 3 to 11 living in agricultural areas of Andalusia, Spain, had undetectable levels of exposure to organophosphate pesticides, with no significant differences observed between agricultural and urban areas.

***Different to our results***

Lu et al., 2000 revealed in a study conducted in the United States that house dust and urinary concentrations of DMP, DMPT, and DMDTP metabolites among agricultural families living in proximity to pesticide-treated orchards were seven times higher than those residing further from orchards but still within the same community. This finding highlights that significant exposure to organophosphate pesticides may not solely result from pathways such as diet, drinking water, and residential pesticide use. Instead, house dust, spray drift, and residues on items like work boots and farmworkers' steering wheels can be noteworthy sources of contamination.

**References**

1. Aelion C. M., and H. T. Davis. "Blood lead levels in children in urban and rural areas: Using multilevel modeling to investigate impacts of gender, race, poverty, and the environment." Science of the Total Environment 694 (2019): 133783.
2. Al-Rajhi M. A., M. R. D. Seaward, and A. S. Al-Aamer. "Metal levels in indoor and outdoor dust in Riyadh, Saudi Arabia." Environment international 22.3 (1996): 315-324.
3. Arcury Thomas A., Haiying Chen, Paul J. Laurienti, Timothy D. Howard, Dana Boyd Barr, Dana C. Mora et al. "Farmworker and nonfarmworker Latino immigrant men in North Carolina have high levels of specific pesticide urinary metabolites." Archives of environmental & occupational health 73.4 (2018): 219-227.
4. Arcury Thomas A., [Haiying Chen](https://www.sciencedirect.com/author/57126555300/haiying-chen), [Sara A. Quandt](https://www.sciencedirect.com/author/7005921858/sara-a-quandt), [Jennifer W. Talton](https://www.sciencedirect.com/author/35338060600/jennifer-w-talton), [Kim A. Anderson](https://www.sciencedirect.com/author/57203051239/kim-a-anderson), Richard P. Scott, et al. "Pesticide exposure among Latinx children: Comparison of children in rural, farmworker and urban, non-farmworker communities." Science of The Total Environment 763 (2021): 144233.
5. Bradman Asa, Lesliam Quirós-Alcalá, Rosemary Castorina, Raul Aguilar Schall, Jose Camach, Nina T Holland et al. "Effect of organic diet intervention on pesticide exposures in young children living in low-income urban and agricultural communities." Environmental health perspectives 123.10 (2015): 1086-1093.
6. Carli Fabrizia, Sabrina Tait, Luca Busani, Demetrio Ciociaro, Veronica Della Latta, Anna Paola Pala et al. "Exposure to Endocrine Disruptors (Di (2-Ethylhexyl) phthalate (DEHP) and Bisphenol A (BPA)) in Women from Different Residing Areas in Italy: Data from the LIFE PERSUADED Project." International Journal of Molecular Sciences 23.24 (2022): 16012.
7. Caruso Rosalie V., Richard J. O'Connor, W. Edryd Stephens, K. Michael Cummings, Geoffrey T. Fong et al. "Toxic metal concentrations in cigarettes obtained from US smokers in 2009: results from the International Tobacco Control (ITC) United States survey cohort." International journal of environmental research and public health 11.1 (2014): 202-217.
8. Cruz Susana, Celeste Lino, and Maria Irene Silveira. "Evaluation of organochlorine pesticide residues in human serum from an urban and cu rural populations in Portugal." Science of the Total Environment 317.1-3 (2003): 23-35.
9. Cutanda Francisco, [Holger M. Koch](https://www.sciencedirect.com/author/35336917300/holger-m-koch), [Marta Esteban](https://www.sciencedirect.com/author/57194740758/marta-esteban-lopez), Jinny Sánchez, [Jürgen Angerer](https://www.sciencedirect.com/author/7102500539/juergen-angerer), [Argelia Castaño](https://www.sciencedirect.com/author/7005340777/argelia-castano) et al. "Urinary levels of eight phthalate metabolites and bisphenol A in mother–child pairs from two Spanish locations." International journal of hygiene and environmental health 218.1 (2015): 47-57.
10. DeLuca N.M., Thomas, K., Mullikin, A. Rachel Slover, Lindsay W. Stanek, Andrew N. Pilant et al. Geographic and demographic variability in serum PFAS concentrations for pregnant women in the United States. J Expo Sci Environ Epidemiol 33, 710–724 (2023). https://doi.org/10.1038/s41370-023-00520-6
11. Elvia Lara F., Harlow D Sioban, Hernández P Bernardo, Sánchez Carrillo Constanza et al. "Organochlorine pesticide exposure in rural and urban areas in Mexico." Journal of Exposure Science & Environmental Epidemiology 10.4 (2000): 394-399.
12. Foxenberg Robert J., Corie A. Ellison, James B. Knaak, Changxing Ma, James R. Olson "Cytochrome P450-specific human PBPK/PD models for the organophosphorus pesticides: chlorpyrifos and parathion." Toxicology 285.1-2 (2011): 57-66.
13. Frederiksen Hanne, Jeanette Kolstrup Søgaard Nielsen, Thit Aarøe Mørck, Pernille Winton Hansen, Janne Fangel Jensen, Ole Nielsen, Anna-Maria Andersson et al. "Urinary excretion of phthalate metabolites, phenols and parabens in rural and urban Danish mother–child pairs." International journal of hygiene and environmental health 216.6 (2013): 772-783.
14. Friberg, Lars. Cadmium in the Environment. CRC press, 2018.
15. Fromme Hermann, Sheryl A. Tittlemier, Wolfgang Völkel, Michael Wilhelm, Dorothee Twardella. "Perfluorinated compounds–exposure assessment for the general population in Western countries." International journal of hygiene and environmental health 212.3 (2009): 239-270.
16. González-Alzaga B., D. Romero-Molina, I. López-Flores, M.J. Giménez-Asensio, A.F. Hernández, M. Lacasaña. "Urinary levels of organophosphate pesticides and predictors of exposure in pre-school and school children living in agricultural and urban communities from south Spain." Environmental research 186 (2020): 109459.
17. Harada K., Nakanishi S, Saito N., Tsutsui T, Koizumi A. "Airborne perfluorooctanoate may be a substantial source contamination in Kyoto area, Japan." Bulletin of environmental contamination and toxicology 74 (2005): 64-69.
18. Harada K., Nakanishi S, Sasaki K, Furuyama K, Nakayama S, Saito N, Yamakawa K et al. "Particle size distribution and respiratory deposition estimates of airborne perfluorooctanoate and perfluorooctanesulfonate in Kyoto area, Japan." Bulletin of environmental contamination and toxicology 76.2 (2006): 306-310.
19. Hrubá Františka, Milena Černá, Chunying Chen, Florencia Harari, Milena Horvat, Kvetoslava Koppová, Andrea Krsková et al. "A regional comparison of children's blood cadmium, lead, and mercury in rural, urban and industrial areas of six European countries, and China, Ecuador, and Morocco." International Journal of Occupational Medicine and Environmental Health 36.3 (2023): 349.
20. Jin Guang-Zhu, Se-Jin Lee, Jung-Ho Kang, Yoon-Seok Chang, Yoon-Young Chang. "Suppressing effect of goethite on PCDD/F and HCB emissions from plastic materials incineration." Chemosphere 70.9 (2008): 1568-1576.
21. Knudsen Lisbeth E., Pernille Winton Hansen, Seher Mizrak, Heidi K. Hansen, Thit A. Mørck, Flemming Nielsen, Volkert Siersma et al. "Biomonitoring of Danish school children and mothers including biomarkers of PBDE and glyphosate." Reviews on environmental health 32.3 (2017): 279-290.
22. Kodavanti P. R. S., J. E. Royland, and KRS Sambasiva Rao. "Toxicology of persistent organic pollutants." (2014).
23. Kuivenhoven Matthew, and Kelly Mason. "Arsenic toxicity." (2019).
24. Laamech Jawhar, Alfred Bernard, Xavier Dumont, Bouchra Benazzouz, Badiaa Lyoussi. "Blood lead, cadmium and mercury among children from urban, industrial and rural areas of Fez Boulemane Region (Morocco): relevant factors and early renal effects." International journal of occupational medicine and environmental health 27 (2014): 641-659.
25. Larsson Kristin, Karin Ljung Björklund, Brita Palm, Maria Wennberg, Lennart Kaj, Christian H. Lindh et al. "Exposure determinants of phthalates, parabens, bisphenol A and triclosan in Swedish mothers and their children." Environment international 73 (2014): 323-333.
26. Li Xu, Xinqi Wang, Yuyan Yang, Yuanduo Zhu, Xu Han, Li Li, et al. "Characteristics, distribution, and children exposure assessment of 13 metals in household dust in China: A big data pilot study." Indoor air 32.1 (2022): e12943.
27. Lu Chensheng, Richard A. Fenske, Nancy J. Simcox, David Kalman. "Pesticide exposure of children in an agricultural community: evidence of household proximity to farmland and take home exposure pathways." Environmental research 84.3 (2000): 290-302.
28. Mørck Thit A., Flemming Nielsen, Jeanette K.S. Nielsen, Volkert D. Siersma, Philippe Grandjean, Lisbeth E. Knudsen "PFAS concentrations in plasma samples from Danish school children and their mothers." Chemosphere 129 (2015): 203-209.
29. Mrema Ezra J., Federico M. Rubino, Gabri Brambilla, Angelo Moretto, Aristidis M. Tsatsakis, Claudio Colosio. "Persistent organochlorinated pesticides and mechanisms of their toxicity." Toxicology 307 (2013): 74-88.
30. Nieboer Evert, Leonard J. S. Tsujib, Ian D. Martinb and Eric N. Liberdac. "Human biomonitoring issues related to lead exposure." Environmental Science: Processes & Impacts 15.10 (2013): 1824-1829.
31. Panieri Emiliano, Katarina Baralic, Danijela Djukic-Cosic, Aleksandra Buha Djordjevic, Luciano Saso. "PFAS molecules: a major concern for the human health and the environment." Toxics 10.2 (2022): 44.
32. Perera Frederica P., V. Rauh, R.M. Whyatt, D. Tang, W.Y. Tsai, J.T. Bernert et al. "A summary of recent findings on birth outcomes and developmental effects of prenatal ETS, PAH, and pesticide exposures." Neurotoxicology 26.4 (2005): 573-587.
33. Rakkestad Kirsten Eline, Christian Jarle Dye, Karl Espen Yttri, Jørn Andreas Holme, Jan Kenneth Hongslo, Per Everhard Schwarze et al. "Phthalate levels in Norwegian indoor air related to particle size fraction." Journal of environmental monitoring 9.12 (2007): 1419-1425.
34. Rudel Ruthann A., and Laura J. Perovich. "Endocrine disrupting chemicals in indoor and outdoor air." Atmospheric Environment 43.1 (2009): 170
35. Runkel Agneta A., Anja Stajnko, Janja Snoj Tratnik, Darja Mazej, Milena Horvat, Petra Přibylová et al. "Exposure of children and adolescents from Northeastern Slovenia to per-and polyfluoroalkyl substances." Chemosphere 321 (2023): 138096.
36. Runkel Agneta A., Darja Mazej, Janja Snoj Tratnik, Žiga Tkalec, Tina Kosjek, Milena Horvat. "Exposure of men and lactating women to environmental phenols, phthalates, and DINCH." Chemosphere 286 (2022): 131858.
37. Runkel Agneta A., Janja Snoj-Tratnik, Darja Mazej, Milena Horvat. "Urinary phthalate concentrations in the slovenian population: An attempt to exposure assessment of family units." Environmental research 186 (2020): 109548
38. Sasaki Kazuaki, Kouji Harada, Norimitsu Saito, Tsuyoshi Tsutsui, Sadahiro Nakanishi, Hideaki Tsuzuki et al. "Impact of airborne perfluorooctane sulfonate on the human body burden and the ecological system." Bulletin of environmental contamination and toxicology 71.2 (2003): 408-413
39. Stöcklin Laura, Georg Loss, Erika von Mutius, Juliane Weber, Jon Genuneit, Elisabeth Horak et al. "Health-related quality of life in rural children living in four European countries: the GABRIEL study." International journal of public health 58 (2013): 355-366.
40. Ventrice Pasquale, Domenica Ventrice, Emilio Russo, Giovambattista De Sarro. "Phthalates: European regulation, chemistry, pharmacokinetic and related toxicity." Environmental toxicology and pharmacology 36.1 (2013): 88-96.
41. Wang Lei, Tianzhen Liu, Fang Liu, Junjie Zhang, Yinghong Wu, Hongwen Sun. "Occurrence and profile characteristics of the pesticide imidacloprid, preservative parabens, and their metabolites in human urine from rural and urban China." Environmental Science & Technology 49.24 (2015): 14633-14640.
42. Wang Yufei, and Haifeng Qian. "Phthalates and their impacts on human health." Healthcare. Vol. 9. No. 5. MDPI, 2021.
43. Wielgomas Bartosz, and Marta Piskunowicz. "Biomonitoring of pyrethroid exposure among rural and urban populations in northern Poland." Chemosphere 93.10 (2013): 2547-2553.
44. Yang Lixin, Yuanyuan Zhang, Feifei Wang, Zidie Luo, Shaojuan Guo, Uwe Strähle. "Toxicity of mercury: Molecular evidence." Chemosphere 245 (2020): 125586.
